# The OÆSE endstation at BESSY II: *operando* X-ray absorption spectroscopy for energy materials

**DOI:** 10.1107/S160057752500116X

**Published:** 2025-03-27

**Authors:** Raul Garcia-Diez, Johannes Frisch, Marianne van der Merwe, Romualdus Enggar Wibowo, Mihaela Gorgoi, Elmar Kataev, Catalina E. Jimenez, Mauricio D. Arce, William Smith, Wilson Quevedo-Garzon, Regan G. Wilks, Dirk Wallacher, Leonhard J. Reinschlüssel, Gülen C. Tok, Hubert A. Gasteiger, Marcus Bär

**Affiliations:** a Interface Design Helmholtz-Zentrum Berlin für Materialien und Energie GmbH (HZB) Albert-Einstein-Straße 15 12489Berlin Germany; b Energy Materials In-Situ Laboratory Berlin (EMIL), HZB, Albert-Einstein-Straße 15, 12489Berlin, Germany; c Departamento de Caracterización de Materiales, Instituto de Nanociencia y Nanotecnología (INN-CNEA-CONICET) Centro Atómico Bariloche Avenida Bustillo 9500 8400Bariloche Argentina; d Sample Environment, HZB, Albert-Einstein-Straße 15, 12489Berlin, Germany; e Chair of Technical Electrochemistry, Department of Chemistry and Catalysis Research Center, TUM School of Natural Sciences Technical University Munich Lichtenbergstraße 4 85748Garching bei München Germany; f Department of Chemistry and Pharmacy Friedrich-Alexander-Universität Erlangen-Nürnberg (FAU) Egerlandstr. 3 91058Erlangen Germany; g Department for X-ray Spectroscopy at Interfaces of Thin Films Helmholtz-Institute Erlangen-Nürnberg for Renewable Energy (HIERN) Albert-Einstein-Str. 15 12489Berlin Germany; Bhabha Atomic Research Centre, India

**Keywords:** X-ray absorption spectroscopy, endstation, *operando* studies, *in situ* studies, electrochemistry, batteries

## Abstract

The investigation of a wide range of energy materials under relevant operation conditions, allowing for real-time investigations of the (electro)chemical mechanisms governing the performance of related applications, is enabled by the new Operando Absorption and Emission Spectroscopy at EMIL (OÆSE) endstation in the Energy Materials In-situ Laboratory Berlin (EMIL) at the BESSY II synchrotron facility in Berlin, Germany. Currently primarily used for X-ray absorption spectroscopy (XAS) studies, the OÆSE endstation utilizes the undulator-based two-colour EMIL beamline (covering an energy range between 80 and 10000 eV) to enable soft, tender, and hard XAS. In this work, the setup, along with *operando* sample environments tailored to address specific questions, is described, emphasizing its modularity and adaptability, and detailing specific strategies to minimize undesired radiation-induced effects caused by the high brilliance of the EMIL beamline. The *in situ* growth of electrodeposited copper monitored by soft and hard XAS, at the Cu *L*_3_ edge (sXAS) and Cu *K* edge (hXAS), respectively, is used as a proof-of-concept experiment, showcasing the capabilities of the OÆSE endstation as a versatile tool for comprehensive *in situ*/*operando* studies of energy materials under relevant conditions.

## Abstract 2

The OÆSE endstation at EMIL at the BESSY II synchrotron facility in Berlin allows real-time investigation of energy materials through *operando*X-ray absorption spectroscopy. The possibility to use soft, tender, and hard X-rays combined with versatile *operando* sample environments enables the study of a wide range of energy materials under relevant operation conditions.

## 1. Introduction

*In situ* studies of promising energy conversion and storage materials under conditions close to real operation are of crucial importance for next-level understanding of the performance-limiting mechanisms of the reactions occurring at (electro)chemical interfaces. *Operando*X-ray spectroscopy has emerged as a powerful technique for investigating the dynamic behaviour of materials and (electro)chemical processes under realistic operating conditions (Chakrabarti *et al.*, 2017 ▸; Crumlin *et al.*, 2015 ▸; Lim *et al.*, 2023 ▸), especially in catalysis research (Varsha & Nageswaran, 2020 ▸; Sarma *et al.*, 2023 ▸; Liu *et al.*, 2020 ▸) and in relevant electrochemical systems, like batteries (Fehse *et al.*, 2021 ▸; Jia *et al.*, 2021 ▸), supercapacitors (Bagge-Hansen *et al.*, 2015 ▸) or fuel cells (Siebel *et al.*, 2016 ▸; Wibowo, Garcia-Diez, Bystron *et al.*, 2023 ▸). In particular, synchrotron-based X-ray absorption spectroscopy (XAS) stands out for its ability to offer detailed insights into the electronic structure of the atomic environments altered during (electro)chemical processes. The atom-specificity and remarkable chemical sensitivity of the method allow the examination of the chemical environment of the probed element, *e.g.* its oxidation states and coordination number, and the quantification of orbital covalency, which are closely related to the underlying mechanisms governing the performance of electrochemical devices (Mendoza *et al.*, 2022 ▸; Bokarev & Kühn, 2020 ▸; Baker *et al.*, 2017 ▸). Recent advances in the design of *operando* sample environments specifically tailored for XAS measurements have significantly broadened the scope of investigations to encompass conditions close to real-world applications and industrially relevant scenarios (Schön *et al.*, 2017 ▸; Velasco-Velez *et al.*, 2017 ▸; Schwanke *et al.*, 2016 ▸; Tesch *et al.*, 2022 ▸). This has enabled the *in situ* probing of crucial solid/liquid or solid/gas interfaces of energy materials relevant for thermal catalysis (Eggart *et al.*, 2021 ▸; Pandit *et al.*, 2022 ▸), electrocatalysis (Timoshenko & Cuenya, 2021 ▸; Rotonnelli *et al.*, 2023 ▸) or batteries (Jia *et al.*, 2021 ▸; Ghigna & Quartarone, 2021 ▸; Gorlin *et al.*, 2015 ▸; Wandt *et al.*, 2016 ▸; Jung *et al.*, 2019 ▸) in real time.

Through the resonant probing of, most commonly, *K* or *L*_2,3_ edges, XAS has been extensively used in both the soft X-ray regime (photon energies lower than ∼2 keV) and the tender/hard X-ray regime (∼2–10 keV) to study energy materials under realistic operating conditions, as illustrated by numerous references in the literature (Jia *et al.*, 2021 ▸; Schön *et al.*, 2017 ▸; Velasco-Velez *et al.*, 2017 ▸; Schwanke *et al.*, 2016 ▸; Tesch *et al.*, 2022 ▸; Eggart *et al.*, 2021 ▸; Pandit *et al.*, 2022 ▸; Timoshenko & Cuenya, 2021 ▸; Rotonnelli *et al.*, 2023 ▸; Ghigna & Quartarone, 2021 ▸; Gorlin *et al.*, 2015 ▸; Wandt *et al.*, 2016 ▸; Jung *et al.*, 2019 ▸). *K*-edge XAS uses the resonant excitation of 1*s* core level electrons to higher-energy unoccupied electronic states of the atom, while *L*_2,3_-edge XAS resonantly excites 2*p* core electrons. Both excitations are governed by the dipole selection rule Δ*l* = ±1, which consequentially determines the symmetry of the unoccupied states that are probed (*K*-edge XAS probes *p*-like unoccupied states; *L*_2,3_-edge XAS probes *s*- or *d*-like unoccupied states), meaning that a combination of the two techniques can provide a more complete mapping of the material’s electronic structure. Although the combination of *K*- and *L*_2,3_-edge XAS has been used to investigate energy materials in operation, benefiting from the complementary information on the electronic structure that can be obtained from these edges (Tsunekawa *et al.*, 2020 ▸; Huang *et al.*, 2022 ▸), the three to four orders of magnitude variation in attenuation length within the soft to tender/hard energy range poses a significant challenge for the design of a common shared sample environment that allows reproducible (electro)chemistry during XAS measurements in the different photon energy ranges. Besides the requirement of the sample environment to be compatible with the high-vacuum needs of soft X-rays, only a few beamlines worldwide currently allow the use of soft and tender/hard X-rays without the need for changing the sample environment (Weinhardt *et al.*, 2021 ▸; Lee & Duncan, 2018 ▸; Reininger *et al.*, 2011 ▸), which can be problematic if *operando* studies require a complex infrastructure, *e.g.* gas supply/detection (and safety measures). For the complementary simultaneous/consecutive collection of XAS at the *K* and *L*_2,3_ edges for elements that are used in energy materials (*e.g.* transition metals), this ability is, however, indispensable. Due to these constraining reasons, an *operando* investigation of energy materials under realistic operating conditions using a combination of soft and tender/hard X-ray absorption spectroscopy in photon-in/photon-out (PIPO) mode in the same experiment has been, to the best of our knowledge, not reported yet, although a few studies focusing on one system studied under operating conditions at different soft, tender and/or hard X-ray beamlines have been reported (Tsunekawa *et al.*, 2020 ▸; Yamamoto *et al.*, 2020 ▸).

For this purpose, in the Energy Materials In-situ Laboratory Berlin (EMIL) at the BESSY II synchrotron facility, we have set up the Operando Absorption and Emission Spectroscopy at EMIL (OÆSE) endstation that allows spectroscopic studies of energy materials under operating conditions with high-brilliance synchrotron radiation. The undulator-based two-colour EMIL beamline provides photon energies tunable between 80 and 10000 eV, allowing XAS measurements at the *K* edge of light elements, *K* and *L*_2,3_ edges of 3*d* transition metals, or *L*_2,3_ and *M*_4,5_ edges of some heavier elements, as will be detailed in Section 2.2. The heart of the OÆSE endstation is its modular and flexible *operando*/*in situ* sample environment, based on *operando* cells that are designed and optimized specifically to address certain research questions using PIPO spectroscopy. The *operando* cells interface with a vacuum analysis chamber *via* a thin X-ray transparent window separating the sample, *e.g.* a cell with a liquid electrolyte under atmospheric pressure, from the analysis chamber in a vacuum and the beamline environment, as will be discussed in Section 2.1. This design allows for modular sample environments, ensuring easy adaptation for various experiments related to energy conversion and storage materials, which will be exemplified in Section 2.3 for cells designed for electrochemistry in flow and static modes. Other aspects surrounding the user experience at OÆSE will be detailed in Section 2.4, including the experiment user interface and data management.

Another important aspect tackled at the OÆSE endstation has been the implementation of measures to mitigate un­desired radiation-induced effects, which can affect the integrity of the obtained spectroscopic data and jeopardize the subsequent conclusions (Weatherup *et al.*, 2018 ▸; Newton *et al.*, 2020 ▸). As presented in Section 3, hardware-based strategies have proven effective towards this issue while ensuring reproducible and accurate measurements in complex liquid-based environments.

In this work, we present the first experimental results obtained at the OÆSE endstation in the fields of electrocatalysis and battery research, showcasing the capabilities of the available sample environments and the two-colour EMIL beamline, while discussing strategies to minimize undesired radiation-induced effects. As a case study, we present the electroplating of copper monitored *in situ* by Cu *L*_3_- and *K*-edge XAS in Section 4, with special focus on the competing deposition mechanisms probed by potentio­dynamic spectroscopy and the unique technical possibilities enabled by the *operando* infrastructure available at OÆSE.

## 2. The OÆSE endstation: *operando* infrastructure and beamline

### 2.1. Design of the sample environment and technical considerations of the analysis chamber

**Figure 1:**
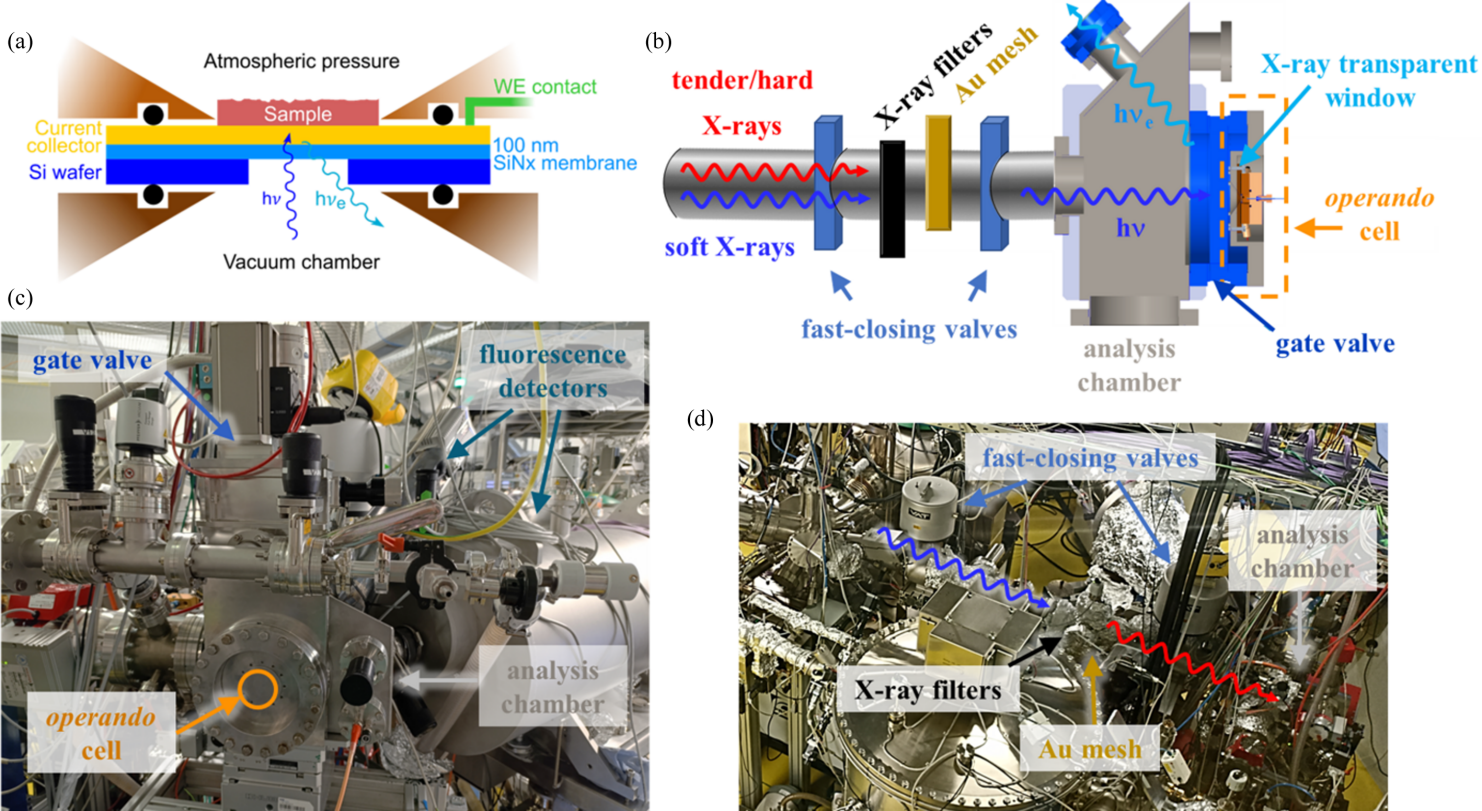
(*a*) Conceptual design of the sample environment built around a thin X-ray transparent window (100 nm thick SiN_*x*_ in this case). (*b*) Scheme of the endstation, including the sample environment, the analysis chamber and the *operando* beamline section. Photographs of (*c*) the analysis chamber and (*d*) the *operando* beamline section, where the most significant components discussed in the text are labelled.

The core of the OÆSE endstation is the modular and versatile *operando*/*in situ* sample environment designed for PIPO spectroscopy studies using soft and tender/hard X-rays. Central to the design is the integration of a thin X-ray transparent window [typically a 50 to 200 nm thick non-stoichiometric silicon nitride (SiN_*x*_) membrane for soft X-rays, and a micrometre-thick Kapton-based foil when tender/hard X-rays are used], strategically positioned to separate the sample from the analysis chamber under vacuum conditions (<1 × 10^−7^ mbar). As shown schematically in Fig. 1 ▸(*a*), the thin and flexible window fulfils two essential functions. Firstly, it enables the transmission of incident and emitted photons with reasonable attenuation, while effectively handling the mechanical stress caused by the pressure difference between the vacuum chamber and the probed environment, at least at atmospheric pressure. Secondly, the window acts as a support structure for the sample under study. Thus, it serves as a substrate for *e.g.* a metal-based current collector necessary for electrochemical measurements, or for photocurrent detection when instead of (or in addition to) the PIPO mode the collection of a total electron yield (TEY) signal is desired.

Some noteworthy aspects of the design of the *operando* measurement setup revolve around the connection between the sample environment and the analysis chamber. Inspired by the design of the SALSA endstation at the Advanced Light Source (Blum *et al.*, 2009 ▸), the *operando* cell of OÆSE is attached to a standard CF150 flange that is connected to a pneumatically actuated gate valve located at the back of the analysis chamber. This allows easy access to all of the important components and connections of the cell from the ambient side. The cell is connected to the analysis chamber through a stainless steel adapter and an O-ring, providing the necessary vacuum tightness [see the sketch in Fig. 1 ▸(*b*)]. After closing the gate valve, a relatively rapid sample exchange is possible, as only the small volume of the chamber containing the cell is vented. This design also significantly reduces the pumping time required to reach the necessary vacuum conditions (essential for conducting soft X-ray spectroscopic measurements) again after sample exchange, which can be completed within 30 minutes (see Section S1 of the supporting information for typical pumping times).

The analysis chamber is mounted on a motorized three-axis stage (Huber *XY*-linear Table 5102.20-X1 and *Z*-stage 5103.A20-40X1) with 10 µm precision and is connected to the fixed portion of the beamline *via* flexible bellows, allowing alignment of the cell *via* positioning of the chamber. This allows mapping of the sample position, enabling the determination of the available area for measurements prior to spectra collection, as well as measuring the material homogeneity, as will be discussed in Section 4.2. The analysis chamber features multiple CF40 flanges pointed at the sample position, allowing multiple detectors to be mounted. The flanges used for the fluorescence detectors are positioned at a 45° angle relative to the incident beam, which impinges normally onto the window [see Fig. 1 ▸(*b*)]; thus, XAS is measured in reflection geometry at OÆSE. The selection of detectors considers the broad range of X-ray energies covered by the EMIL beamline and the requirements for different detection modes based on the experiment’s needs. Total fluorescence yield (TFY) mode generally offers bulk-sensitive XAS with the highest count rates, while partial fluorescence yield (PFY) mode provides element sensitivity (see Section S2 of the supporting information for a comparison of both detection modes on the Cu *L*_3_ edge of an electrodeposited Cu material on a Pt-coated 100 nm SiN_*x*_ window). The detectors currently utilized in the analysis chamber of OÆSE are listed in Table 1 ▸. The additional TEY detection mode collected by the sample drain current allows for a higher surface sensitivity, as in this case the probing depth is determined by the inelastic mean free path of the outgoing electrons.

### 2.2. EMIL beamline and *operando* beamline section

The OÆSE endstation is designed to operate on the high-brilliance undulator-based two-colour EMIL beamline. The beamline sources are two canted undulators: the soft X-ray UE48 and the cryogenic in-vacuum U17 for hard X-rays, which are both in-house developments. Energy tunability and monochromatization are achieved with a plane grating monochromator (PGM, 800 lines mm^−1^ and 400 lines mm^−1^) in the soft X-ray range of 80 to 2300 eV and with a DCM [double-crystal monochromator, Si(111), Si(311) and Si(422) pairs] in the hard X-ray range of 2 to 10 keV. Minimum foci sizes at the OÆSE sample position are 123 µm × 14 µm (U17) and 105 µm × 20 µm (UE48), with a flux on the order of 10^12^ photons s^−1^ and energy resolutions of 0.1 eV at 400 eV, 0.2 eV at 2500 eV and 0.44 eV at 4500 eV at standard settings (Follath *et al.*, 2013 ▸; Hendel *et al.*, 2016 ▸; Gorgoi, M. *et al.*, in preparation ▸). Currently, both EMIL branch lines offer photon energy scans in stepping mode, with the soft X-ray branch also allowing for scans in continuous mode, in which the monochromator moves constantly, enabling a faster energy scan than the stepping mode.

As depicted in Fig. 1 ▸(*b*), OÆSE is connected to the EMIL beamline, downstream of the SISSY I endstation designed for the study of solid-state samples (van der Merwe *et al.*, 2023 ▸), by a beamline section that includes elements to monitor the beamline’s flux (semi-transparent gold mesh with *in situ* Au-evaporation possibility) and X-ray filters that allow a reduction of the photon flux dose impinging on the sample (if required), as will be further detailed in Section 3.2 when discussing strategies to mitigate radiation-induced effects. Protection measures against potential pressure bursts in the event of window ruptures have also been implemented, utilizing two fast-closing valves (10 ms shutting times, VAT 75.0 fast-closing valves) located upstream of OÆSE, as observed in the photographs in Figs. 1 ▸(*c*) and 1 ▸(*d*).

### 2.3. Sample environments for *operando* electrochemistry studies

The sample environments share a common modular design, enabling easy adaptation to specific experimental requirements when studying different energy materials under operating conditions. Due to the continuously growing catalogue of *operando*/*in situ* cells sharing a common basic design, here we will only present the sample environments devoted to electro­chemistry in a liquid environment currently available on OÆSE. These electrochemical cells allow the study of the critical solid/liquid electrode|electrolyte interface, enabled by the thin X-ray transparent window that serves as both separator and substrate for the deposition of the working electrode (WE) and/or the WE current collector (WE CC), as presented in Fig. 1 ▸(*a*).

#### 2.3.1. Flow-through cell with elevated temperature option

**Figure 2:**
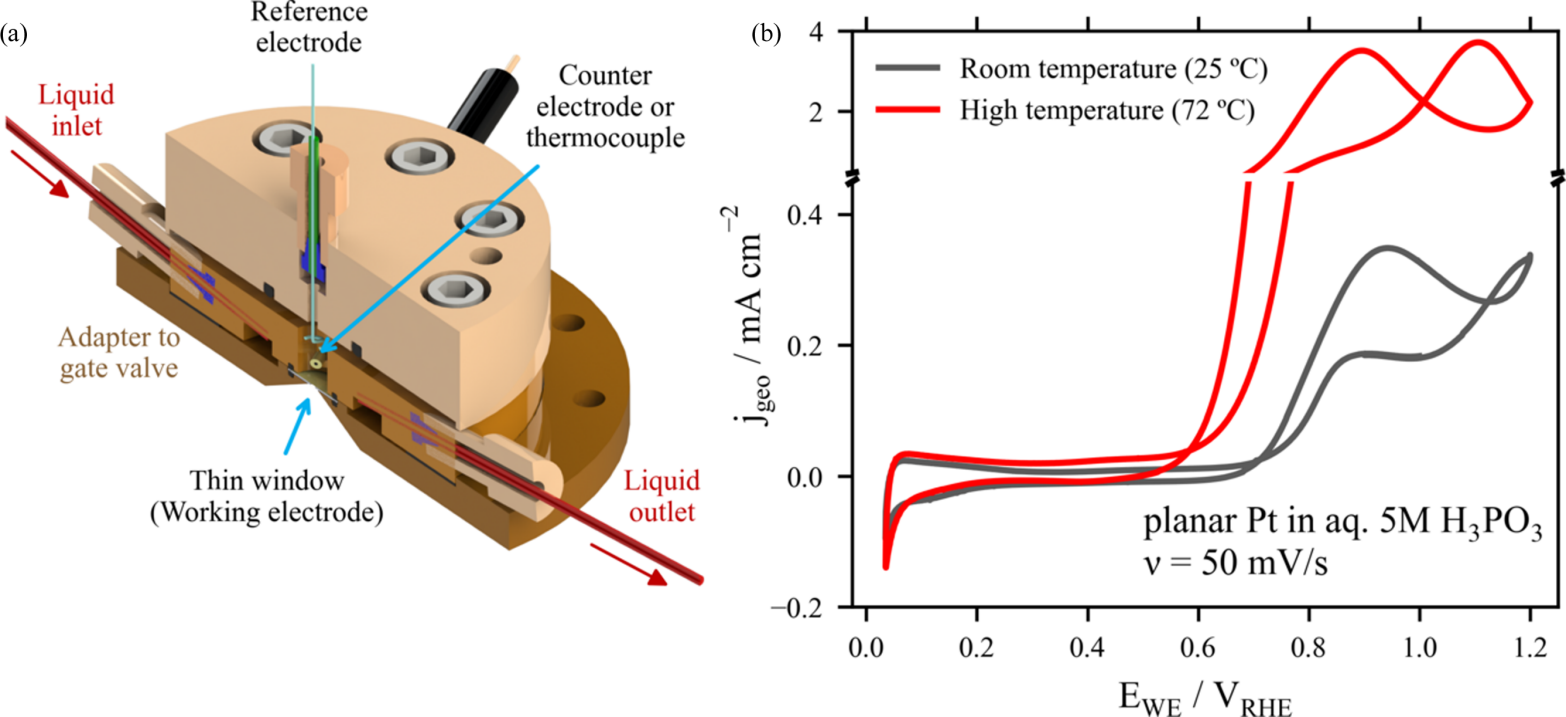
(*a*) Schematic presentation of the three-electrode flow-through *operando* cell. (*b*) Operation of the cell at different temperatures: cyclic voltammograms measured at room temperature and 72°C of a planar Pt WE in aq. 5 *M* H_3_PO_3_ electrolyte using a Pt wire as CE and an RHE as RE.

This cell provides reliable and reproducible three-electrode electrochemistry thanks to its well designed reactor volume and the corrosion resistant materials used, like the polyetheretherketone (PEEK) used for the cell’s parts and flange­less liquid connections, which provide chemically inert pathways. As depicted in Fig. 2 ▸(*a*), the three-part modular design of this cell and its accessibility from the ambient side ensure quick and straightforward exchange of the liquid connections and of the counter electrode (CE) and reference electrode (RE), even during operation.

The experimental setup also supports a continuous-flow configuration to give precise control of the electrolyte flow during electrochemical measurements in the 0.75 ml cell volume. Tunable flow is achieved through a syringe pump operating in push–pull mode, enabling flow rates ranging from 0.01 to 12500 µl min^−1^. The adjustable flow control facilitates efficient mass transport for different electrochemical reactions and minimizes beam irradiation effects, as the electrolyte is continuously replenished and possible radiolysis products are removed from the probed volume.

The cell enables measurements at moderately elevated temperatures, *i.e.* up to 90°C for aqueous electrolytes. This is achieved by heatable tubing and heating pads at the PEEK cell, while the temperature is monitored in the reactor volume, close to the probed sample position on the thin window, as depicted in Fig. 2 ▸(*a*) (see the scheme of the complete sample environment indicating the measurement of temperature at different relevant positions in Section S3 of the supporting information). This allows the study of the temperature dependence of the electrochemical activity, being a key feature for studying materials under relevant operating conditions, which for electrochemical devices in many cases implies temperatures above room temperature (Shi *et al.*, 2022 ▸). In the example presented in Fig. 2 ▸(*b*), a 15 nm planar Pt WE sputtered on a 12 µm Kapton foil (with a 5 nm thick Ti adhesion layer) is studied in aqueous 5 *M* H_3_PO_3_ electrolyte [CE: Pt wire, RE: RHE (reverse hydrogen electrode)] at room temperature and at 72°C. As expected, at elevated temperature the electrochemical oxidation of H_3_PO_3_ is enhanced (Prokop *et al.*, 2015 ▸), as illustrated by the roughly tenfold increase in the current density *j*_geo_ in the region of *E*_WE_ > 0.6 V versus RHE in the cyclic voltammogram recorded at 72°C compared with that recorded under room-temperature conditions. As described in the P *K*-edge XAS study of the oxidation behaviour of aqueous H_3_PO_3_ (Wibowo *et al.*, 2024 ▸), increased oxidation of the electrolyte at high temperatures is crucial for the performance of high-temperature proton-exchange membrane fuel cells (HT-PEMFC).

#### 2.3.2. Static cell

In electrochemical experiments that are susceptible to trace impurities in the liquid phase, *i.e.* the electrolyte, the use of a continuous-flow cell would lead to the sustained occurrence of parasitic side reactions. For instance, in battery systems, a multitude of undesired reactions can be triggered by trace impurities such as alcoholates or HF: gas formation through their oxidative (at the positive electrode) or reductive (at the negative electrode) decomposition, etching of the cathode active material, and reaction with the solid electrolyte interphase (SEI) (Jung *et al.*, 2019 ▸; Huang *et al.*, 2022 ▸; Gallus *et al.*, 2014 ▸; Freiberg *et al.*, 2020 ▸). To minimize the extent of these reactions, a low and static amount of electrolyte should be used in these fields of research. Additionally, in order to obtain reliable results, the *operando* cells employed to investigate these systems should show comparable electrochemical performance to cell setups that are being used on a laboratory scale, *e.g.* coin cells. One further challenge for *operando* cells arises with regard to the controlled cell stack pressure (*e.g.* ∼0.2 MPa in pouch or coin cells), which is required to achieve homogeneous cell compression, prevent electrode delamination and lower electrochemical impedance (Cao *et al.*, 2022 ▸). Hence, reproducibility and control of the operational pressures on the thin X-ray transparent window of an *operando* cell are essential to obtain an overall electrochemical performance that is comparable with laboratory-scale cells.

**Figure 3:**
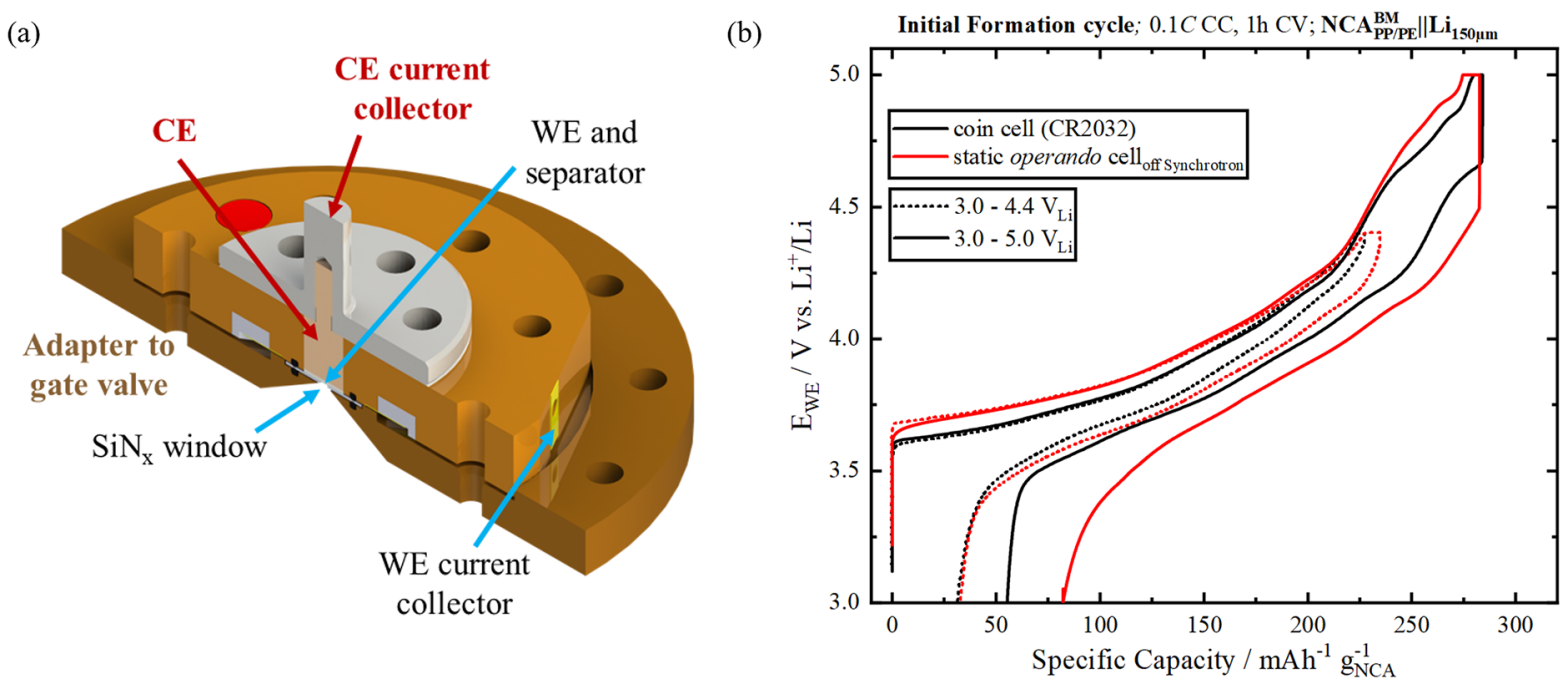
Static *operando* cell. (*a*) Cross-sectional drawing of the two-electrode static cell. (*b*) Comparison of the static cell first charge and discharge performance to different upper cut-off voltages (red lines) with that obtained with a conventional coin cell (black lines). The static cell contains a working electrode composed of primary crystallites of LiNi_0.80_Co_0.15_Al_0.05_O_2_ (NCA) coated on a polypropyl­ene separator, two glass-fibre separators, a lithium metal anode and 5 µl of LP57 (1 *M* LiPF_6_ in 3:7 EC:EMC) electrolyte. The working electrode material is pressed onto a 100 nm SiN_*x*_ window coated with a 100 nm Al film serving as current collector. The coin cell was assembled with the same electrodes and separators, adding 90 µl of the same electrolyte. The cells were charged at a rate of 0.1 C (based on the NCA theoretical capacity of 279 mA h cm^−2^) to 4.4 V_Li_ (dashed lines) or 5.0 V_Li_ (solid lines), where the potential was held for 1 h or until the current dropped below 0.05 C, before the cells were discharged to 3.0 V_Li_.

These two challenges can be overcome on the OÆSE endstation through the usage of an *operando* static cell design, as depicted in Fig. 3 ▸(*a*). The two-part modular design enables precise control of the internal pressure by the usage of cell parts machined with narrow tolerances. By carefully adapting the length of the CE pin, the thicknesses of the electrodes and the separator, and the amount of electrolyte used, the stack pressure applied onto the thin X-ray transparent window (SiN_*x*_, 100 nm thickness, Silson, UK) can be precisely controlled. The usage of perfluorinated O-ring sealings (FFKM, Angst + Pfister, Germany) ensures an air- and vacuum-tight environment while minimizing the diffusive loss of the organic electrolyte solvents.

As for the above-described flow-through cell, the static cell is accessible from the ambient side of the OÆSE endstation, enabling rapid exchange of cells if required.

Fig. 3 ▸(*b*) shows an example of the electrochemical performance of the static cell. These *operando* cells were assembled with a working electrode (WE, 5 mm diameter) based on primary particles of LiNi_0.80_Co_0.15_Al_0.05_O_2_ (denoted NCA; BASF TODA Battery Materials LLC, Japan) coated onto a polypropyl­ene substrate (5 mm diameter, FS2190, Freudenberg Group, Germany) with two glass fibre separators (6 mm diameter, VWR, Germany), with a lithium metal disc (75 µm thickness, 5 mm diameter, Chemetall Foote Corporation, USA) as CE, and with 5 µl 1 *M* LiPF_6_ in EC:EMC (EC, ethylene carbonate; EMC, ethyl methyl carbonate, LP57, Gotion, USA) as electrolyte. To provide current collection from the WE in the cell, the SiN_*x*_ window is coated with a 100 nm thick layer of aluminium. See Section S3 of the supporting information for further details of the cell assembly.

The charge and discharge performance with different upper cut-off potentials (4.4 V_Li_ as dotted lines, and 5.0 V_Li_ as solid lines) of the static cell (data plotted in red) is compared with the performance obtained in a conventional coin cell (CR2032, Hohsen, Japan, data plotted in black), which was assembled with the same materials as described above. It has to be noted here that the static cells presented herein were cycled in an Ar-filled glovebox and were not attached to the OÆSE endstation. As can be seen in Fig. 3 ▸(*b*), the static cell performance for the first cycle between 3.0 and 4.4 V_Li_ (red solid line) conducted at a low rate of 0.1 C is in quite good agreement with that of a conventional coin cell (black solid line), demonstrating the suitability of the novel static cell design for *operando* spectroscopic experiments.

The developed cell design is not only limited to the study of cathode active materials for batteries [used as a WE that is placed near the X-ray window, as in Fig. 3 ▸(*b*)]. Anode active materials such as lithium metal, silicon or graphite can also be used as a WE, which can give insights into the complex formation of the SEI (Swallow *et al.*, 2022 ▸). Tracking of the chemical states evolving during supercapacitor operation might give insights into the reasons for their degradation.

### 2.4. Experiment user interface, remote access, and data management

The availability of experimental time at OÆSE *via* periodic ‘calls for proposals’ from the synchrotron facility BESSY II allows external users to make use of the *operando* capabilities described above. To improve the user experience, an important focus lies in facilitating the interface of the users with the experiment, typically consisting of different layers of complexity. The devices of the OÆSE endstation and the EMIL beamline are controlled through dedicated Graphical User Interfaces (‘user panels’ or GUIs) that allow for easy access to the configuration of the different detectors and beamline elements necessary to optimize the spectroscopic measurement. In parallel, data from all relevant devices are recorded and archived, and the history of variables such as the pressure of the vacuum chamber, upstream photon fluxes, temperature of the photon detectors or the optical elements in the beamline can all be plotted in real time. The OÆSE endstation also allows for remote access, allowing control of the most relevant elements of the experiment, *e.g.* the sample position, the potentiostat or the configuration of the detectors. This is achieved by the remote desktop tool *NoMachine* (https://www.nomachine.com) operated from an external machine using a URL link (valid for the duration of the allocated experimental campaign).

The open-source Python tool *Bluesky* (Allan *et al.*, 2019 ▸) is responsible for the orchestration of the experimental protocols, communicating with devices *viaEPICS*. *Bluesky* allows the user to perform custom-made protocols necessary to accomplish, for example, strategies to mitigate radiation-induced effects (see Section 3) or automated variations in the experimental conditions (*e.g.* temperature, applied potential *etc.*). Currently, through an IPython command-line tool, *Bluesky* allows for flexible approaches to measurement protocols, ranging from the high complexity accessible by experienced users to standard established routines. The measurement results are plotted live, extending the agency of the user to decide *ad hoc* about the integrity of a measurement, while data from previous measurements can be browsed and plotted in *PyMca* (Solé *et al.*, 2007 ▸). Additionally, a ‘jupyter hub’ is provided which serves *Jupyter Notebooks* to users. In these notebooks, common scientific Python packages are installed and users are provided with example scripts for additional processing and analysis.

For enhanced accessibility, the measurement data are automatically exported at the end of every measurement to .csv, *specfiles* and *NeXus* files and, in an effort for completeness and traceability, files include all motors and the configuration parameters of every device on the EMIL beamline and in the *operando* infrastructure, as well as extensive metadata of the experiment. At the end of the user’s experimental campaign, the measurement files are exported to a remote file share, also accessible externally.

## 3. Strategies to mitigate radiation-induced effects during XAS measurements

An important aspect to consider when performing measurements in liquid-based environments with synchrotron radiation from a high-brilliance undulator source like the EMIL beamline is the possible radiation-induced damage that can lead to erroneous data interpretation. Though the resonant excitation of the probed elements at their atomic absorption edges during XAS is a unique tool to enhance chemical sensitivity, it also increases the susceptibility to radiation-induced damage, especially in the soft and hard X-ray regimes (Zabilska *et al.*, 2022 ▸). Photoelectrochemical effects are often observed in liquid-based environments and can be caused by direct ionization of the sample by X-rays or by indirect means, *e.g.* by radiolysis of the reactant, solvent or electrolyte (Albrahim *et al.*, 2021 ▸), as observed in some electrochemical systems (Weatherup *et al.*, 2018 ▸; King *et al.*, 2019 ▸) including aqueous electrolytes, where water radiolysis leads to the generation of radicals that can adversely affect the probed sample (Le Caër, 2011 ▸; Gopakumar *et al.*, 2023 ▸; Unger *et al.*, 2017 ▸).

The OÆSE endstation incorporates special measures to identify and minimize radiation-induced damage during *in situ*/*operando* XAS measurements on (electrified) liquid/solid interfaces, aiming to maintain high signal-to-noise ratios while reducing the incident X-ray dose without compromising on other aspects of the probing light, like focal size and position, resolving power or collimation.

In the following, we differentiate the mitigation strategies specially built into the *operando* infrastructure detailed in Sections 2.1 and 2.2 from those approaches arising from the (de)tuning of the optical elements and the insertion device. Typically, a combination of complementary strategies arises as the best experimental solution.

### 3.1. Measures implemented in the *operando* infrastructure

#### 3.1.1. Attenuation filters

A motorized stage with a selection of filters provides the possibility of adjusting the flux of the incident beam, *i.e.* the X-ray dose, for each experiment. A set of standard filters have been chosen to ensure enough attenuation at the different photon energy ranges available on the endstation: three Kapton-based filters with 12, 24 and 36 µm thicknesses are utilized for the tender/hard X-ray regime, while an Ir/SiC filter (40 nm of Ir on 100 nm of SiC) and an SiN_*x*_ (1000 nm) filter are employed complementarily to allow for measurements in the soft X-ray regime (see the calculated attenuations of the different filters in Section S4 of the supporting information). The filter stage allows for the easy addition of new filters to adapt to diverse experimental requirements or the exchange of degraded ones. Potential degradation of the filters over time can be identified through changes in the measured intensity, monitored by the gold mesh.

Importantly, the measurement of the photocurrent behind the filters by the gold mesh precisely determines the photon flux at the sample position, as depicted in Fig. 1 ▸(*b*). This allows for accurate normalization of the acquired data and ensures the reliability of the spectroscopic measurements. A thermal evaporation system allows the periodic preparation of a clean Au surface.

#### 3.1.2. Beam blocking between data acquisition

**Figure 4:**
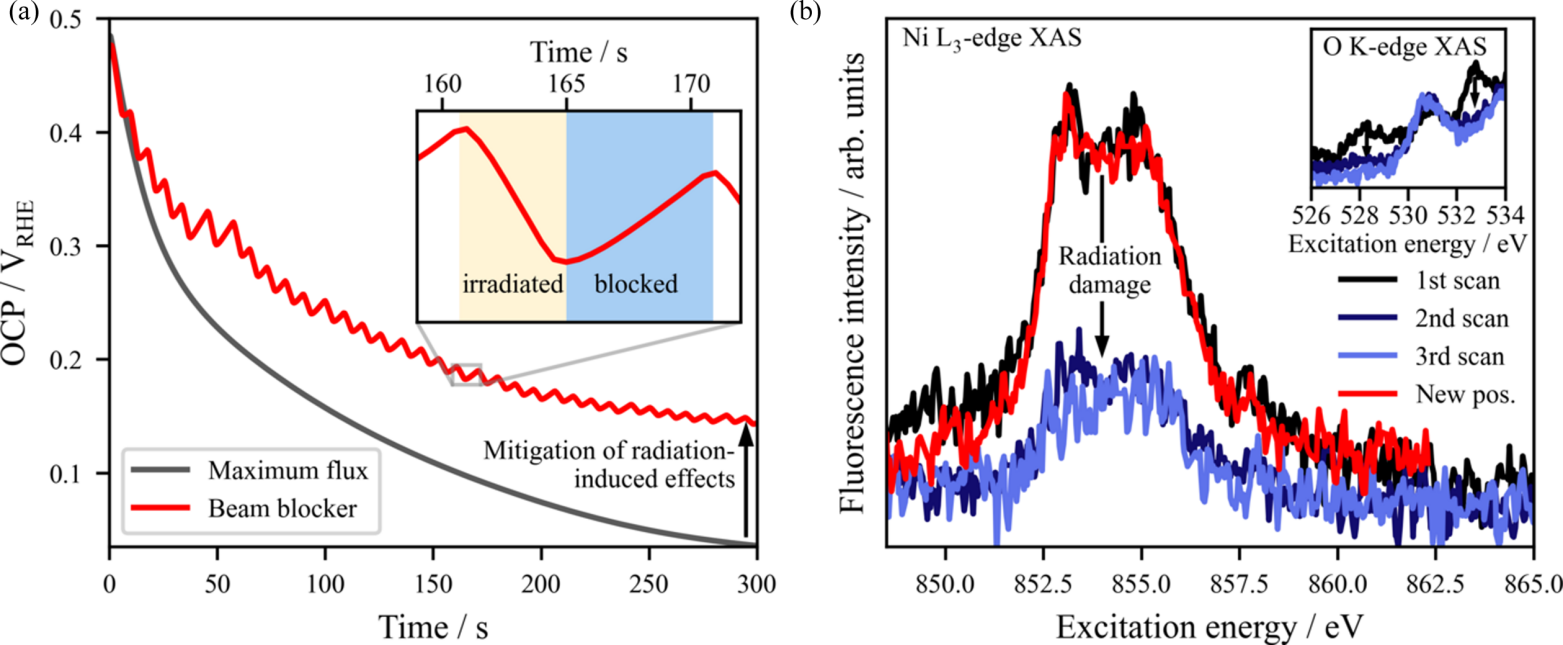
Strategies implemented in the *operando* infrastructure to mitigate radiation-induced effects. (*a*) Beam blocking between data acquisition. The radiation-induced oxidation of H_3_PO_3_ electrolyte in the presence of a thin Pt electrode, indicated by the decrease in the OCP (due to H_2_ generation), is minimized by a valve opening only during effective measurement times. (*b*) Example of a measurement system that requires a variation in the position of the irradiated sample, showing the alteration of the Ni *L*_3_-edge spectroscopic fingerprint of the NCA WE detailed in Section 2.3 (and at the O *K* edge, shown in the inset) when being irradiated three times consecutively at the same position, and the subsequent recovery of the spectroscopic signal when probing at a new sample spot that had not yet been exposed to the X-ray beam.

In XAS, dead times between the actual data acquisition can result from the necessary movement of the monochromator and from the read-out times of detectors and monitoring devices, leading to unnecessary irradiation of the probed sample. This prolonged radiation exposure can enhance undesirable radiation-induced effects without contributing to the collected spectroscopic signal. To mitigate this issue and reduce the absolute radiation dose received by the sample during dead times, a beam-blocking valve that opens only during data acquisition is installed upstream in the EMIL beamline before the elements shown in Fig. 1 ▸(*b*). The positive effects of this strategy have been demonstrated in the electro­chemical flow-through cell, where the open circuit potential (OCP) of a 15 nm thick Pt film WE on a Kapton film in the presence of aqueous 5 *M* H_3_PO_3_ is significantly affected by radiation with photons having an energy of 2160 eV [resonant excitation at the P *K* edge, 7 × 10^11^ photons s^−1^ as measured by a photodiode (AXUV100G, Optodiode) located in front of the sample position] due to the generation of H_2_ arising from water radiolysis and the radiation-induced oxidation of phospho­rous acid (H_3_P^3+^O_3_) to phospho­ric acid (H_3_P^5+^O_4_) (Wibowo *et al.*, 2024 ▸). The use of this beam blocker during dead times translates into a 57% reduction in the total radiation dose on the sample. As presented in Fig. 4 ▸(*a*), this strategy results in the recovery of the system’s OCP during dead times (related to the slower production of H_2_, a by­product of the radiation-enhanced oxidation of H_3_PO_3_) and ensures that the investigated system is closer to application-relevant conditions. This approach proves to be highly beneficial for experiments requiring long monochromator movement between measurement points or for detectors operating in close-to-saturation modes with prolonged read-out times.

#### 3.1.3. Variation in the position of the irradiated sample

The motorized analysis chamber interfacing with the sample environment facilitates the systematic variation of the sample’s position during consecutive measurements (10 µm precision), minimizing the radiation dose received by a given sample spot. Considering the small beam profile detailed in Section 2.2, a multitude of different spots within the available sample area can be measured without overlap of the irradiated regions, significantly extending the probed sample’s lifetime and enhancing the data reliability. Combined with a continuous liquid flow in the *operando* cell, this strategy has demonstrated remarkable efficiency in mitigating radiation-induced effects (Wibowo *et al.*, 2024 ▸).

Beyond the flow-through cell, this approach has also proven valuable in the static cell design described in Section 2.3. An example of this strategy is illustrated in Fig. 4 ▸(*b*), where an NCA cathode active material studied in the two-electrode static cell shows an irreversible alteration of its spectroscopic signature at the Ni *L*_3_ and the O *K* edges when irradiated consecutively at the same position. This undesired change in the absorption features arises from the, probably radiation-induced, polymerization of the carbonate-based electrolyte (Gupta *et al.*, 2020 ▸; Kusumoto *et al.*, 2020 ▸), which changes the local density of the system, thus increasing the X-ray attenuation. This can be observed in the decrease in the electrolyte-related peak intensity at around 528 eV and 533 eV in the O *K*-edge XAS [see inset of Fig. 4 ▸(*b*)], as well as in the overall decrease of the Ni spectroscopic signal due to the higher number of primary and fluorescence photons arising from the NCA cathode being reabsorbed in the polymerized system. The radiation-induced effect significantly diminishes the signal-to-noise ratio of the spectroscopic fingerprint of the probed NCA material, prompting the need for a mitigation strategy to address these challenges effectively. As shown by the recovery of the Ni *L*_3_-edge signal taken on a spot of the sample that had not yet been exposed to the X-ray beam [red line in Fig. 4 ▸(*b*)], changing the sample position prior to the acquisition of each spectrum clearly mitigates this issue.

### 3.2. Adjusting the beamline configuration

**Figure 5:**
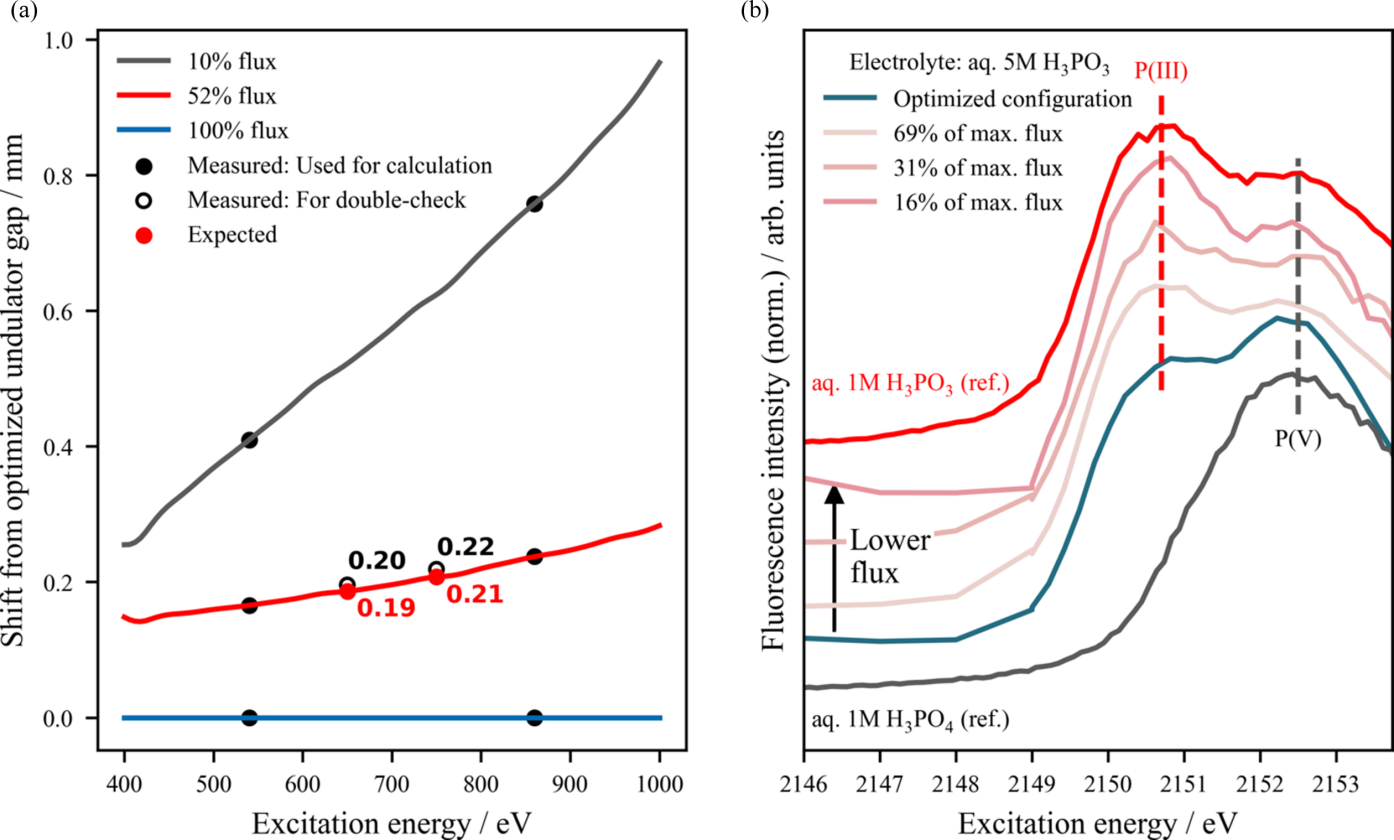
Strategies based on (de)tuning the insertion device, *i.e.* undulator gap, to mitigate radiation-induced effects. (*a*) Predicted undulator gap in the soft X-ray regime (800 lines mm^−1^ grating and third undulator harmonic) required to obtain maximum photon flux (100%, optimized undulator gap configuration), 52% flux and 10% flux of the optimized beam. In order to double-check the energy extrapolation beyond 540 and 860 eV, the undulator gap is detuned to obtain 52% of the maximum flux at 650 and 750 eV. The required gap values (black empty circles), 25.06 mm for 650 eV and 26.91 mm for 750 eV, are shifted 0.20 mm and 0.22 mm, respectively, from the optimized undulator gap configuration (orange line, 100% flux) and only deviate ∼5% from the values predicted using this approach (red circles, 25.05 mm and 26.90 mm, respectively). More details about the undulator gap value as a function of energy and photon flux can be found in Section S5 of the supporting information. (*b*) In the planar Pt | H_3_PO_3_ electrolyte system, P *K*-edge XAS measurements using the U17 DCM show a clear radiation-induced oxidation of the H_3_P^3+^O_3_ electrolyte at high photon fluxes. The highest conversion rate of P^3+^ to P^5+^ species is observed with the maximum flux configuration (optimized beam), where the spectral feature at 2152.5 eV attributed to H_3_P^5+^O_4_ dominates over the H_3_P^3+^O_3_ signal at 2150.8 eV (Wibowo, Garcia-Diez, van der Merwe *et al.*, 2023 ▸). The reduction in the photon flux by detuning the undulator gap minimizes the radiation-induced oxidation until the desired situation at 16% of maximum flux, where the obtained spectrum resembles the one obtained on the 1 *M* aq. H_3_P^3+^O_3_ reference.

The photon flux at the sample can be decreased by detuning the respective undulator harmonic (by varying the undulator gap) of the beamline relative to the monochromator energy. The flux emerging from different undulator configurations can be predicted as shown in Fig. 5 ▸(*a*), where the relationship between the photon flux and the undulator gap (UE48 soft X-ray undulator) is depicted for a wide range of photon energies and three different fluxes (10%, 52% and 100% of the maximum flux available, *i.e.* optimized undulator gap configuration). The undulator gap values predicted to halve the flux at the sample’s position were corroborated by measurements at 650 and 750 eV using the 800 lines mm^−1^ UE48 PGM and the third undulator’s harmonic, as shown in Fig. 5 ▸(*a*). More details of the calculation of the flux at the sample position as a function of the detuning of the undulator gap are described in Section S5 of the supporting information.

The Pt | H_3_PO_3_ system previously described gives a good example of the effects of flux reduction *via* undulator detuning due to the rapid radiation-induced oxidation of phospho­rous acid (H_3_P^3+^O_3_) to phospho­ric acid (H_3_P^5+^O_4_) at high fluxes. Fig. 5 ▸(*b*) illustrates the correlation between the photon flux and the oxidation state of phospho­rus in the aqueous electro­lyte in the flow-through cell, where it is evident that employing a less brilliant photon beam results in a reduction in the undesired oxidation of the H_3_PO_3_ electrolyte, as indicated by the change in the ratio between the spectroscopic intensity at 2150.8 eV and 2152.5 eV of the P *K* edge, attributed to P^3+^ and P^5+^ species, respectively [speciation indicated by the reference compounds displayed in Fig. 5 ▸(*b*) based on the work of Wibowo, Garcia-Diez, van der Merwe *et al.* (2023 ▸), where each spectrum is collected at a new sample position after fresh electrolyte is flowed into the cell].

Adjusting the flux of the photon beam effectively mitigates radiation-induced damage and preserves the integrity of the experimental data, though it can come at the expense of the spectroscopic signal-to-noise ratio or the beam characteristics. For instance, a similar reduction in photon flux can be approached by detuning the *cff* parameter (constant focal distance), though the flux cannot be minimized as effectively as by detuning the undulator device (as detailed in Section S6 of the supporting information) and can affect the beamline resolving power at specific configurations.

## 4. Showcase of the OÆSE endstation capabilities: *in situ* study of copper electrodeposition *via* combined soft and hard X-ray PIPO spectroscopies

In this section, we present a proof-of-concept experiment featuring most of the unique technical capabilities of the OÆSE endstation introduced in previous sections; for instance, the sample environment specifically designed for aqueous electrochemistry, spectra collection by both partial fluorescence yield (PFY) and total fluorescence yield (TFY) detectors, and the motorization of the sample which enables micrometre-precise positioning.

The electrodeposition of copper is relatively well understood (Velasco-Vélez *et al.*, 2018 ▸) and therefore is chosen as an appropriate example to demonstrate the capabilities of the experimental facility. By controlling the electroplating process in real time, we can tailor the deposited mass under a certain set of experimental conditions, effectively optimizing the X-ray intensity attenuation of the system, a critical factor in limiting the signal-to-noise ratio, especially in the soft X-ray regime, without compromising the amount of active electrode/electrolyte interface (more details in Section S7 of the supporting information). The selection of a transition metal like copper as the central probed element allows collection of both soft and hard X-ray absorption spectra at the *L*_2,3_ and *K* edges, respectively, offering complementary insights into the material’s electronic configuration and chemical bonding, and distinct probing depths.

The experimental setup involves the three-electrode flow-through *operando* cell presented in Section 2.3 with a 15 nm Pt WE deposited onto a 75 nm SiN_*x*_ window (Silson, UK) using DC magnetron sputtering, in which 5 nm of Ti was used as an adhesion interlayer to enhance the quality of the produced Pt thin film. A Pt wire as CE and Ag/AgCl (silver–silver chloride, SSC) or RHE as reference electrodes were used, while the reaction was carried out in an aqueous 0.1 *M* CuSO_4_ electrolyte. The calibration of the excitation energy was performed following the protocol described in Section S9 of the supporting information.

To elucidate the range of morphological and chemical changes arising from different electrodeposition conditions which can influence the structure of the deposited material onto the Pt WE (Rosa-Ortiz *et al.*, 2019 ▸; Grujicic & Pesic, 2002 ▸), we explored various methods of electrodeposition growth. These encompassed investigating the impact of reduction potential and variations in the adopted electrochemical protocol.

### 4.1. Chemical and structural study of electrodeposited copper by soft and hard XAS

**Figure 6:**
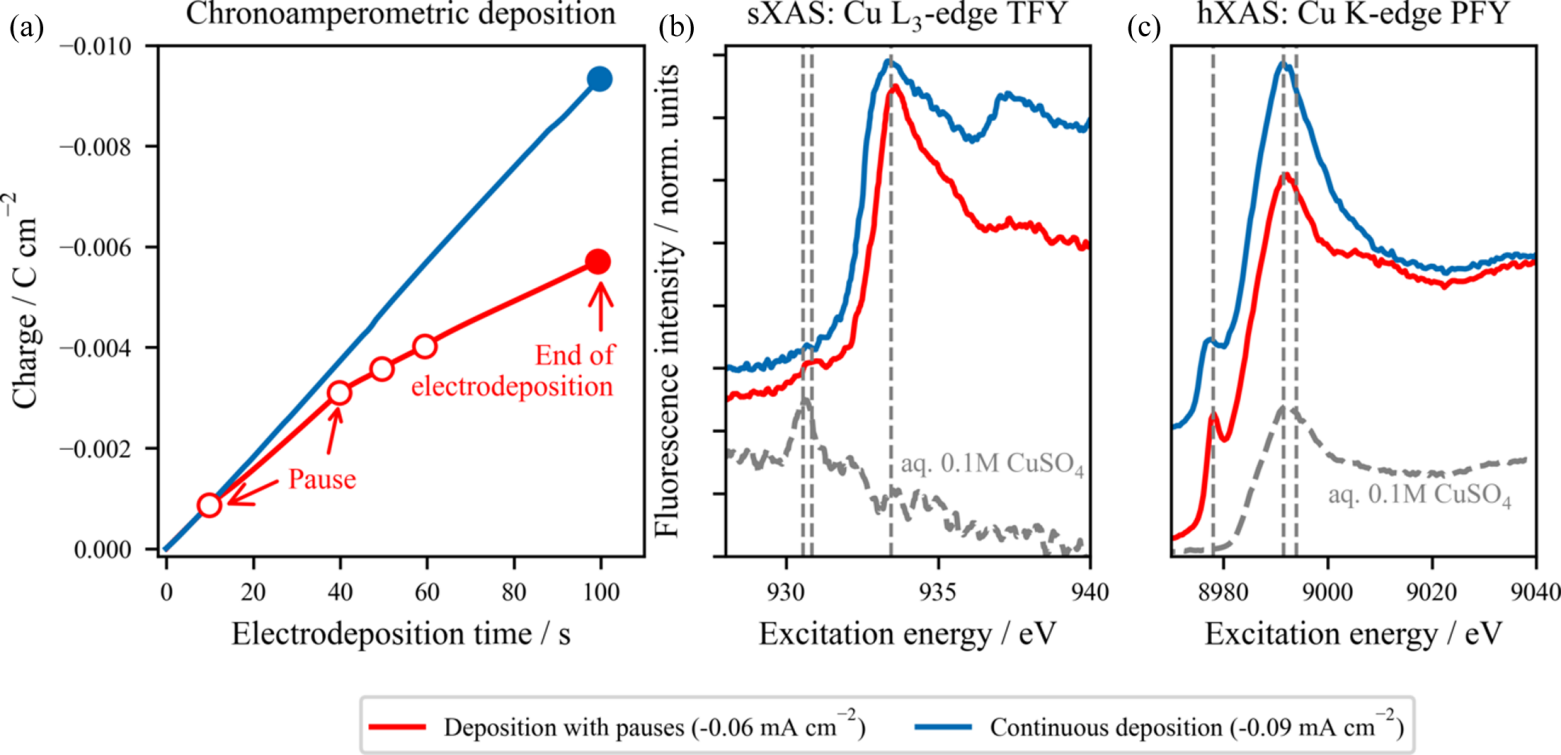
Cu samples electrodeposited under different conditions. (*a*) Chronocoulometric profiles of *in situ* grown Cu materials, deposited continuously (blue) and including intermittent potential application, labelled ‘deposition with pauses’ (red). (*b*) Soft XAS (sXAS) at the Cu *L*_3_ edge in TFY mode and (*c*) hard XAS (hXAS) at the Cu *K* edge in PFY mode (centred at the Cu *L*_α_ emission line) of the two Cu materials. For comparison, the spectrum of the Pt WE prior to electroplating under OCP conditions with a 0.1 *M* CuSO_4_ electrolyte is depicted as a dashed grey line. The vertical dashed lines show the energies related to the Cu(I) and Cu(II) oxides and CuSO_4_.

Fig. 6 ▸ depicts the complementary XAS results obtained at the Cu *L*_3_ and *K* edges of two different Cu samples electrodeposited by applying a current at a constant reductive potential of −0.01 V_RHE_ during 100 s. The chronocoulometric profiles of the two *in situ* grown materials are shown in Fig. 6 ▸(*a*). In one case, continuous potential is applied during the whole electrodeposition treatment (labelled ‘Continuous deposition’), while for the sample labelled ‘Deposition with pauses’, the effect that intermittent potential application can have on both the morphology and chemical composition of electrodeposited copper is studied with pauses of 30 s in OCP, *i.e.* without applied potential. The average current densities 〈*j*_geo_〉 of the process with pauses and by continuous deposition are −0.06 mA cm^−2^ and −0.09 mA cm^−2^, respectively (more information on the current density profiles of the growth can be found in Section S11 of the supporting information).

In Figs. 6 ▸(*b*) and 6 ▸(*c*), the Cu *L*_3_- and *K*-edge X-ray absorption spectra of the two electrodeposited samples are shown, together with the reference spectrum of aqueous 0.1 *M* CuSO_4_, which serves as a background spectrum representing the status before electrodeposition and as an energy reference for a Cu^II^ species. In the sXAS measurements, notable differences arise between the electrodeposition with and without pauses, particularly in the spectroscopic features after the main peak of the Cu *L*_3_-edge at 933.8 eV (Weatherup *et al.*, 2018 ▸; Velasco-Vélez *et al.*, 2018 ▸), which distinguishes the Cu metal (‘Continuous deposition’) and Cu_2_O oxide (‘Deposition with pauses’) materials. The formation of a stable Cu_2_O solid has been previously reported (Velasco-Vélez *et al.*, 2018 ▸), though a two-step deposition process with a Cu^+^-based intermediate has also been reported (Ghosh *et al.*, 2019 ▸). The presence of the oxide only when pauses are introduced in the electrodeposition process is well aligned with the result observed in the electrodeposited material presented in Section S7 of the supporting information, where the incorporation of OCP interruptions enables partial oxidation of the material. This observation suggests that the metallic phase is probably the preferred nucleation species under these reductive conditions.

Although differences in the hard X-ray regime are less evident, the ‘Deposition with pauses’ conditions show a much sharper pre-peak in the Cu *K*-edge hXAS (Larsson *et al.*, 2017 ▸; Chandarak *et al.*, 2011 ▸; Guda *et al.*, 2021 ▸) related to the presence of Cu_2_O in both the bulk and the surface of the electrodeposited material. The hXAS measurement of the ‘Continuous deposition’ sample is in good agreement with the spectral fingerprint of metallic copper (Larsson *et al.*, 2017 ▸; Chandarak *et al.*, 2011 ▸; Guda *et al.*, 2021 ▸), further suggesting the favourable growth of fully reduced Cu under these mild conditions.

The presence of the same chemical species in both Cu *L*_3_- and *K*-edge XAS, jointly with the complementary surface and bulk sensitivity of this technique, points to a homogenous chemical composition throughout the entire electrodeposited material within the depth sensitivity of the method.

### 4.2. Morphological reconstruction of the electrodeposited material with resonant Cu *L*_2,3_ spectromicroscopy

During the electrodeposition of copper, nucleation and seeded growth play crucial roles that significantly influence the chemical state of the resulting material, as discussed above, and, in some cases, its morphology. The location and nature of nucleation sites can lead to the formation of heterogeneous films with incohesive or partial coverages, as well as different characteristic structures, ranging from crystallites or aggregates to thin films or microbeads. In fact, the copper materials electrodeposited by the two growth modes studied above show very different morphologies, as revealed by scanning electron microscopy (SEM) measurements presented in Section S12 of the supporting information. While the ‘Deposition with pauses’ condition generates disconnected Cu_2_O structures ranging between 20 and 30 µm in size, the electrodeposition process without interruption forms a rather cohesive network of ∼1 µm crystallites decorated by smaller structures, hinting at the generation of a more homogeneous current distribution in the WE when the current is continuously passed. The average coverage of Cu on the 15 nm Pt electrode also strongly depends on the electrodeposition process.

**Figure 7:**
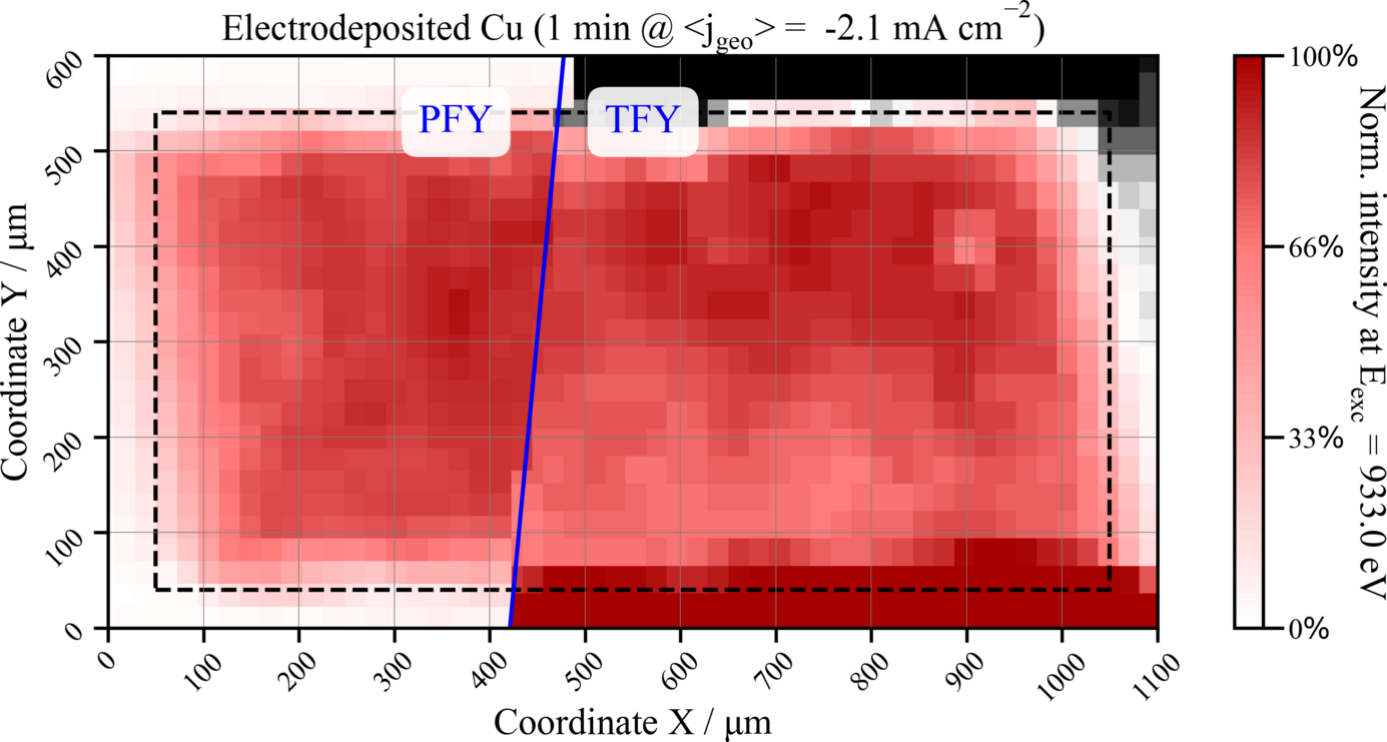
Spectromicrograph of copper electrodeposited during 1 min under very reductive conditions obtained with a Cu *L*_3_-edge resonant excitation energy of 933.0 eV. The right half of the sample was recorded in TFY mode and the left half was collected in Cu *L*_α_ PFY mode. The dashed line marks the shape of the 1000 µm × 500 µm SiN membrane within the Si frame.

The OÆSE endstation’s translation stage allows precise positioning of the sample environment with 10 µm precision, enabling the acquisition of spectromicroscopic images. By resonantly exciting the sample at different photon energies associated with various copper species, highly sensitive insights into the morphology and speciation of the grown material can be obtained *in situ*. As an example, Fig. 7 ▸ presents the spectromicrograph of an electrodeposited Cu material grown during 1 min under very reductive conditions (〈*j*_geo_〉 = −2.1 mA cm^−2^) on a 1000 µm × 500 µm window with a 100 nm SiN_*x*_ membrane (Silson, UK) coated with 15 nm of Pt. Obtained with the resonant excitation energy corresponding to the maximum spectral intensity of both the metallic Cu and Cu_2_O species in the Cu *L*_2,3_-edge spectrum (933.0 eV), the right half of the sample was recorded in TFY mode with a photodiode, while the left half was collected in PFY mode with a silicon drift detector centred at the Cu *L*_α_ emission line.

The spectromicrograph unveils the profound impact of the growth process on the micro-structural characteristics of the material. An overtly heterogeneous topology is discernible across the 1000 µm × 500 µm surface of the electrode (demarcated by dashed lines in Fig. 7 ▸), directly linked with the partial coverage of copper under these specific electrodeposition conditions. This incohesive nature is corroborated by SEM measurements, as illustrated in Section S12 of the supporting information for a material deposited under analogous conditions. The heterogeneity exhibited in the electrodeposited Cu underscores the crucial role of nucleation and seeding in shaping the material’s structure and morphology. These findings complement the insights gained from XAS regarding the diverse chemical species resulting from Cu grown under distinct electrochemical conditions. Nevertheless, due to limitations in the precision of the sample stage and, notably, the beam size (>50 µm), definitive assertions about the dimensions of the sample’s grains or crystallites – typically smaller than 30 µm – cannot be conclusively made.

Additionally, the spectromicrograph in Fig. 7 ▸ illustrates the basic difference between detecting the sample’s photons in PFY mode (left half) and TFY mode (right half). In PFY mode, the detector selectively filters out scattered or reflected photons that do not align with the energies of the selected characteristic emission line (Cu *L*_α_ in this example). This is evident in the photon intensities observed in TFY mode at *y* coordinates >550 µm, where a diminished intensity, probably related to shadowing, is discernible, and at *y* < 100 µm, where the reflections on the sides of the window dominate the signal. The combined use of a resonant excitation energy to amplify the fluorescence arising from the probed sample with the detection of only atom-specific photons by PFY constitutes a robust approach for sample mapping and topological characterization.

### 4.3. Revealing the competing mechanisms of Cu electrodeposition and dissolution using atom-specific FEXRAV under operating conditions

**Figure 8:**
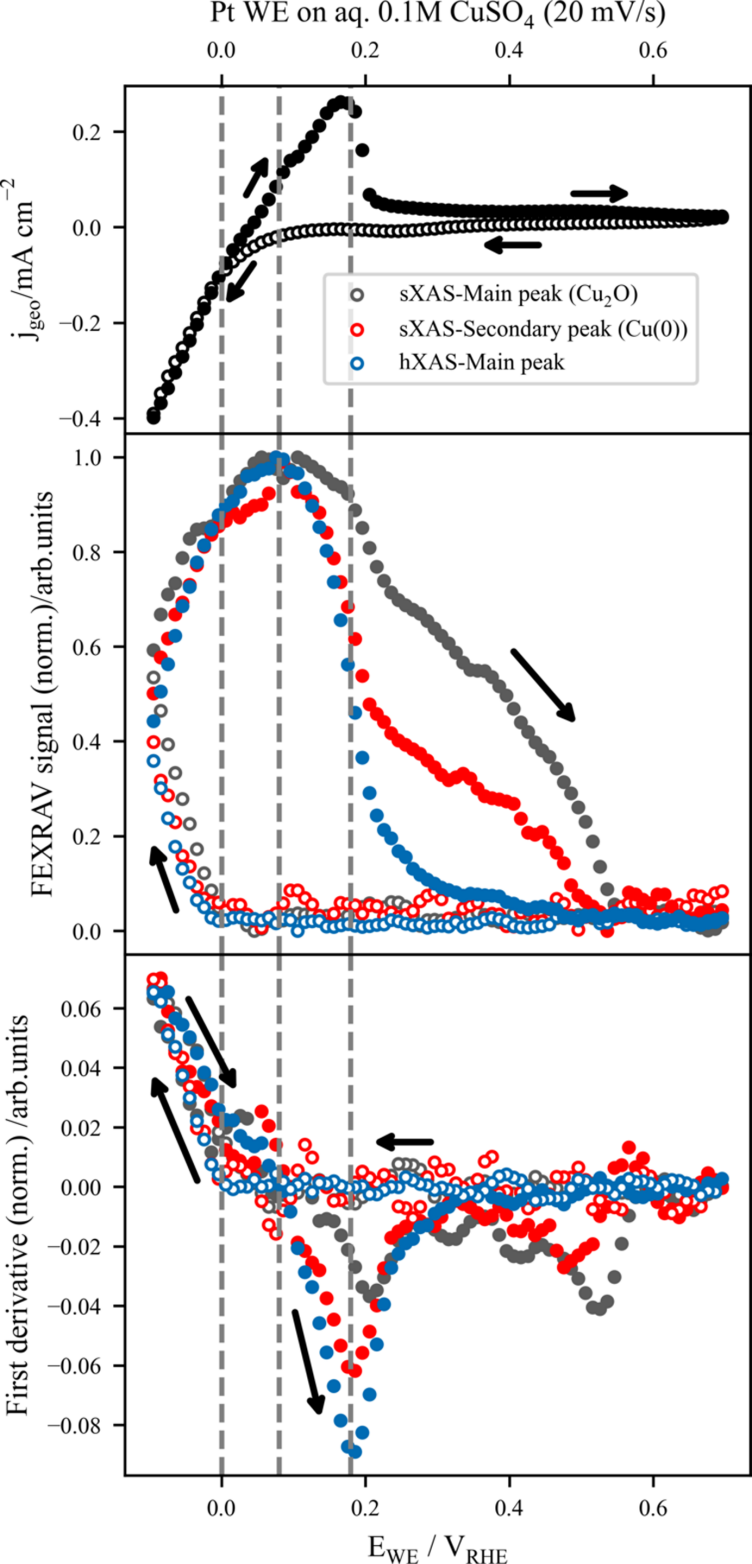
FEXRAV results at the Cu *L*_3_ and *K* edges probing *in situ* the electrodeposition and dissolution mechanism of Cu in aq. 0.1 *M* CuSO_4_. The top panel presents the typical CV of the Pt | aq. 0.1 *M* CuSO_4_ electrochemical system. The FEXRAV signals at three different excitation energies are presented in the middle panel, while the corresponding first derivatives are depicted in the bottom panel. Vertical dashed lines are shown as indicators of the *E*_WE_ at which the relevant electrochemical reactions discussed in the text take place.

As detailed in Section S7 of the supporting information, the characteristic cyclic voltammogram (CV) of the Pt | CuSO_4_ electrochemical system (shown in the top panel of Fig. 8 ▸) possesses two main operating regions: at potentials below ∼0 V_SSC_, a reduction phase involves the electroplating of cupric ions in the electrolyte, while an oxidizing region is observed for applied potentials higher than ∼0 V_SSC_, where the solid copper film dissolves back to solvated Cu^2+^ ions. Therefore, an understanding of the dynamic behaviour during voltammetry is required to comprehend the competing mechanisms during the electrodeposition and oxidation processes of an aqueous copper system.

Fixed-energy X-ray absorption voltammetry (FEXRAV), also known as X-ray spectroelectrochemistry (Tesch *et al.*, 2022 ▸) or potentiodynamic spectroscopy (Frevel *et al.*, 2019 ▸), is a powerful technique employed to gain atom-specific insights into the electrodeposition and dissolution mechanisms of copper *in situ*, with a focus on copper atoms from both the surface and the bulk of the material. This method utilizes XAS to probe selectively the different Cu species during the redox processes and has been shown to be an efficient tool to characterize electrocatalysts during operating conditions, especially in the hard X-ray range (González-Flores *et al.*, 2018 ▸; Pasquini *et al.*, 2021 ▸).

By collecting the fluorescence signal arising from the photoabsorption process at a specific excitation energy as a function of the applied potential *E*_WE_, the different chemical species related to the redox features appearing in the CV can be reconstructed. The excitation energies are chosen to be associated with a distinct chemical species in order to deconvolute the contribution of each species to the process. Based on the hXAS results presented in Fig. 6 ▸(*c*), the main peak energy at 8992.1 eV has been selected for the hard X-ray regime as a bulk-sensitive probe of the electrodeposited material. In the soft X-ray range, derived from the observation presented in Fig. 6 ▸(*b*), the secondary spectral feature associated with Cu metal at 937.8 eV has been used as well as the main peak at 933.8 eV, which correlates with both the presence of Cu metal and Cu^I^ species like Cu_2_O. Fortunately, the lower background of Cu_2_O at 937.8 eV enables discrimination between metallic Cu and Cu^I^ species (Weatherup *et al.*, 2018 ▸) and is used to deconvolute the participation of this species in the CV of the Pt | aq. 0.1 *M* CuSO_4_ electrochemical system presented in Fig. 8 ▸.

In Fig. 8 ▸, the FEXRAV results are presented accompanied by a typical CV of the system recorded at 20 mV s^−1^, where the direction of the potential sweep is indicated by arrows. The three FEXRAV signals depicted in the middle panel of Fig. 8 ▸ show a general trend associated with the two main regions of the CV: an increase in the signal at potentials <0 V_RHE_ in the anodic branch (*E*_WE_ is swept in the positive direction) associated with the reduction phase (Cu electrodeposition), and a decrease in the cathodic branch at >0.08 V_RHE_ related to the dissolution of the previously deposited copper in the oxidizing region. The first derivative in the bottom panel of Fig. 8 ▸ generally shows a maximum at the lowest *E*_WE_, corresponding to the maximum rate of Cu plating, and a minimum at around 0.18 V_RHE_, related to the maximum dissolution of Cu. As expected, the latter has a direct correlation with the highest oxidative peak in the anodic branch of the CV shown in the top panel of Fig. 8 ▸. As previously reported (Tesch *et al.*, 2022 ▸), the extrema observed in the FEXRAV first derivative are a good descriptor of the redox couples present in cyclic voltammetry and can be used to predict the maximum rate of oxidation/reduction of a chemical species.

The non-negligible differences between the FEXRAV signals indicate complex behaviour during electrodeposition and dissolution. For instance, the disappearance of the hXAS signal in the cathodic branch at lower potentials than its sXAS counterparts suggests a lower sensitivity of hXAS at low equivalent Cu thicknesses due to its longer attenuation length. More interestingly, the FEXRAV signal measured with *E*_exc_ = 933.8 eV associated with the Cu_2_O species decreases much more slowly than the Cu^0^-related signal during dissolution, suggesting that oxidized Cu tends to dissolve at higher potentials than metallic copper. In fact, the increase in the Cu_2_O signal in the anodic branch also occurs slightly before that of the metallic species and suggests the favoured presence of Cu^I^ species in this environment. This supports the previous findings about the electrodeposition of Cu under intermittent potential application, where Cu_2_O was observed generated by the partial oxidation of electrodeposited Cu enhanced in an aqueous CuSO_4_ medium. Besides, the first derivative of the Cu^I^ signal shows a minimum at >0.2 V_RHE_, further supporting the observation that higher potentials are required to obtain a maximum dissolution rate of Cu_2_O in comparison with Cu metal, in line with the increasing bond strength and thus stability of Cu—O compared with Cu—Cu.

In the first derivative of the soft X-ray signals, similar minima are observed at more oxidizing potentials (0.50 and 0.55 V_RHE_ for Cu^0^ and Cu_2_O species, respectively). These minima are probably associated with the less energetically favourable desorption of the first Cu layer strongly bound to the Pt electrode, only observable by the more surface-sensitive sXAS.

These findings highlight the importance of combining spectroscopic methods like FEXRAV with electrochemical techniques, in which the deconvolution of the different chemical species can be challenging under certain conditions (Toparli *et al.*, 2017 ▸).

## 5. Conclusions

This work presents the newly developed Operando Absorption and Emission Spectroscopy at EMIL (OÆSE) end­station, established in the Energy Materials In-situ Laboratory Berlin (EMIL) at the synchrotron facility BESSY II in Berlin, Germany. Devoted to photon-in/photon-out X-ray absorption spectroscopy (XAS) studies of energy materials under operation conditions, the OÆSE endstation utilizes the brilliant X-ray photons arising from the undulator-based two-colour EMIL beamline (80 to 10000 eV) for complementary soft and hard XAS investigations, for example, *L*_2,3_- and *K*-edge spectra of transition metals. The *operando* cells used at the OÆSE endstation employ a thin X-ray transparent window to separate the sample from the vacuum environment and are based on a common modular design, ensuring adaptability to diverse energy materials and experimental conditions. In this work, the *operando* electrochemical cells designed for liquid environments have been discussed, highlighting the flow-through cell for temperature-controlled electrocatalytic studies and the static cell for battery research. Both cells allow reproducible electrochemistry, and the static cell shows a comparable performance to coin cells.

Beyond the description of the infrastructure, the risk of radiation-induced damage arising from the use of high-brilliance synchrotron radiation has been tackled extensively in this work, especially when studying systems with aqueous electrolytes where water radiolysis in the soft and tender X-ray regime is probable. The OÆSE endstation implements several strategies to address these challenges, like those measures specially built into the *operando* infrastructure to minimize the total dose impinging on the sample spot (*e.g.* beam blocking between data acquisition, X-ray filters or continuous change of sample position) or related to the detuning of the optimized beamline parameters.

To showcase further the capabilities of the OÆSE end­station, the electrodeposition of copper in an aqueous CuSO_4_ electrolyte was investigated *in situvia* combined soft and hard X-ray photon-in/photon-out spectroscopies. The chemical sensitivity of Cu *L*_3_-edge sXAS was demonstrated by monitoring *in situ* the growth of electroplated Cu and Cu_2_O in the electrochemical *operando* flow-through cell, where insights into both the chemical structure and the equivalent amount of deposited Cu were provided at different stages of the electro­deposition. By virtue of the wide range of energies available on the two-colour EMIL beamline, Cu *L*_3_-edge sXAS and *K*-edge hXAS revealed the chemical species associated with the distinct nucleation and growth processes occurring under different electrodeposition conditions. The complementary penetrating power of sXAS and hXAS allowed a characterization with more sensitivity towards the surface and the bulk of the electrodeposited materials, showing a homogeneous chemical composition. The morphology and topology of the Cu materials was studied by Cu *L*_3_-edge resonant spectromicrography, illustrating the capacity of the OÆSE endstation to map the electrode with very high chemical sensitivity and relatively high spatial resolution (∼50 µm).

Finally, the coupling of electrochemical methods with spectroscopic techniques, *i.e.* FEXRAV in the soft and hard X-ray regime, allowed a reconstruction of the behaviour of the contribution of each species participating in the dissolution of Cu in the Pt | aq. 0.1 *M* CuSO_4_ system during cyclic voltammetry.

In conclusion, this case study exemplifies the capabilities of the OÆSE endstation, allowing the integration of spectroscopic and electrochemical techniques, providing valuable insights into dynamic electrochemical processes and demonstrating its potential for advancing the understanding of complex electrochemical systems.

## 6. Related literature

The following references, not cited in the main body of the paper, have been cited in the supporting information: Celante & Freitas (2010 ▸); Chang *et al.* (1999 ▸); Kamel *et al.* (2022 ▸); Liu *et al.* (2010 ▸); Naikoo *et al.* (2022 ▸); Owens *et al.* (2002 ▸).

## Supplementary Material

Additional information. DOI: 10.1107/S160057752500116X/ye5057sup1.pdf

Authors contributions. DOI: 10.1107/S160057752500116X/ye5057sup2.xlsx

## Figures and Tables

**Table 1:** Fluorescence detectors currently installed in the analysis chamber of OÆSE

	Energy range	Detection mode	Limitations	Technical details
Photodiode	80–10000 eV	TFY	Low quantum efficiency	AXUV 100 G (Optiodiode)
Channeltron	80–10000 eV	TFY	Also collects charged particles	Channel electron multiplier (Dr Sjuts Optotechnik GmbH)
Silicon drift detector	700–10000 eV	PFY and TFY	Limited energy resolution	Bruker XFlash 6|30
Soft X-ray emission spectrometer	50–1800 eV	PFY and TFY	Limited energy range	High transmission soft X-ray emission spectrometer (in-house design in collaboration with JPE, Netherlands)

## Data Availability

The data that support the findings of this study are available within the article and its supporting information. The raw data has been deposited online in a Zenodo repository with the following permanent DOI: https://doi.org/10.5281/zenodo.10866536
