# Supplementary material for: The OÆSE endstation at BESSY II: operando X-ray absorption spectroscopy for energy materials
Source: J Synchrotron Radiat. 2025 Mar 27;32(Pt 3):634–48. doi: 10.1107/S160057752500116X (PMC12067322; doi:10.1107/S160057752500116X)
Supplement: Supplementary file 1 [file s-32-00634-sup1.pdf]

JOURNAL OF SYNCHROTRON RADIATION

**Volume 32 (2025)**

**Supporting information for article:**

# The OAESE endstation at BESSY II: *operando* X-ray absorption spectroscopy for energy materials

**Raul Garcia-Diez, Johannes Frisch, Marianne van der Merwe, Romualdus Enggar Wibowo, Mihaela Gorgoi, Elmar Kataev, Catalina E. Jimenez, Mauricio D. Arce, William Smith, Wilson Quevedo-Garzon, Regan G. Wilks, Dirk Wallacher, Leonhard J. Reinschlüssel, Gülen C. Tok, Hubert A. Gasteiger and Marcus Bär**

### S1. Typical pumping times of the sample environment compartment

Number of events = 19 samples measured in a period of 5 days

Median (dashed line in the plot) = 33.4 minutes to evacuate the sample environment compartment, i.e. to reach measurement conditions (<8e$^{-6}$ mbar)

Mean = 35.0 minutes to evacuate the sample environment compartment, i.e. to reach measurement conditions (<8e$^{-6}$ mbar)

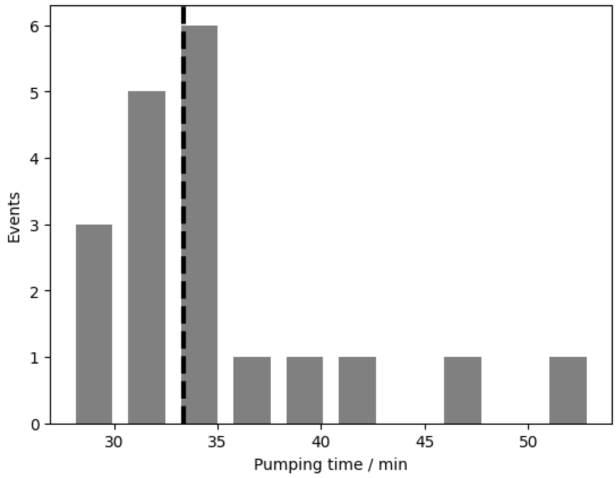


**Figure S1** Pumping time required to reach the necessary vacuum conditions in the sample environment compartment in a sample-set with 19 events.

### S2. Partial Fluorescence Yield (PFY) and Total Fluorescence Yield (TFY) with an energy dispersive detector at the Cu $L_3$-edge

*Operando* XAS measurements use a Si-based window like $SiN_x$ to separate UHV from the probed sample. The window can contribute to the overall spectroscopic signal when probing the Si or the N K-edge of your sample (elements present in the window itself), or when higher order photons excite resonantly the Si K-edge of the window, as discussed below.

In **Figure S2a**, the Si K-edge of the $SiN_x$ window in 2nd order (in red) has a strong spectroscopic signal just below the $L_3$-edge of Cu, which can strongly affect the background of the Cu signal of the electrodeposited material on the Pt working electrode (in green) in TFY mode.

As shown in **Figure S2b**, an energy-dispersive fluorescence detector (Silicon Drift Detector) allows measurements in PFY mode, which enables the deconvolution of the Cu signal related to the electrodeposited copper on the Pt working electrode (Cu $L_\alpha$ emission line) from the Si signal arising from the $SiN_x$ window (Si $K_\alpha$ emission line), improving the signal to noise ratio of the Cu $L_3$-edge XAS.

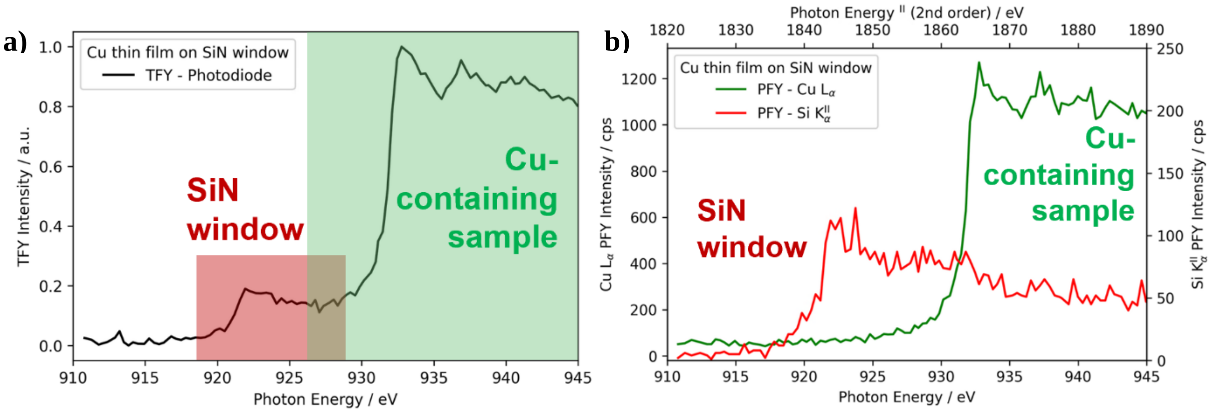


**Figure S2** Cu $L_3$-edge XAS measurement of a Cu material electrodeposited onto a SiNx window coated with Pt as WE in **a)** TFY mode and in **b)** PFY mode.

The PFY mode enhances the chemical sensitivity of XAS and allows for atom-selective fluorescence collection, for instance, by detecting Cu $L_\alpha$ emission photons when exciting resonantly at the Cu $L_3$-edge.

## S3. Expanded details of the sample environments for *operando* electrochemistry studies

### S3.1. Three-electrode flow-cell for elevated temperatures

**Figure S3** illustrates schematically the 3-electrode flow-through *operando* cell with elevated temperature option. Depicted are the different heating elements (heatable tubing and heating pads) as well as the temperature monitors (PFA-coated thermocouples, CASS-IM15G-300-PFA, Omega Engineering, Germany). Most relevantly, the $T_3$-thermocouple monitors the temperature inside the reactor chamber, i.e. close to the probed sample surface. For completeness, the liquid insertion devices (syringe pumps in push-pull mode in this case) are also shown.

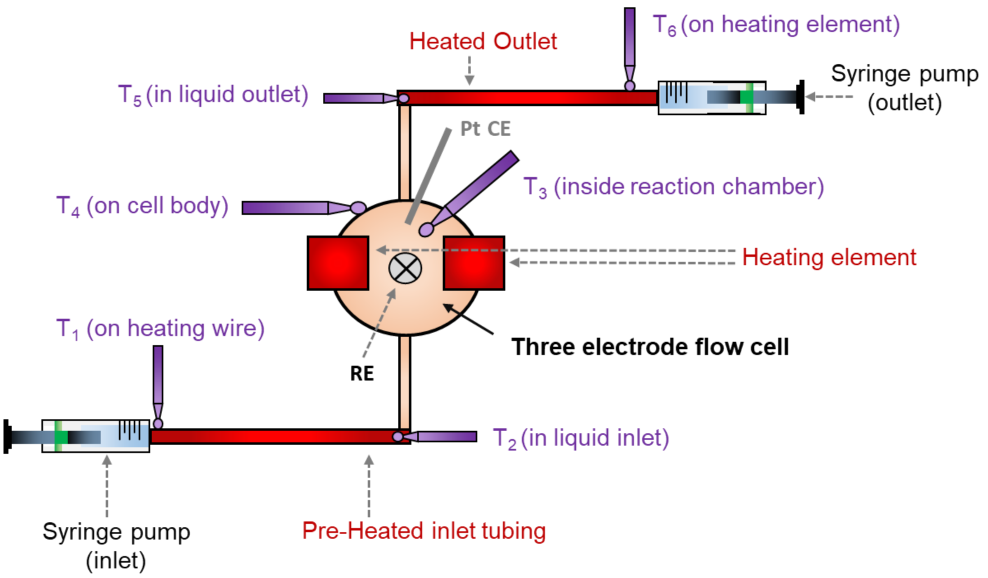


**Figure S3** Schematic presentation of the 3-electrode flow-through operando cell with elevated temperature option.

### S3.2. Static cell

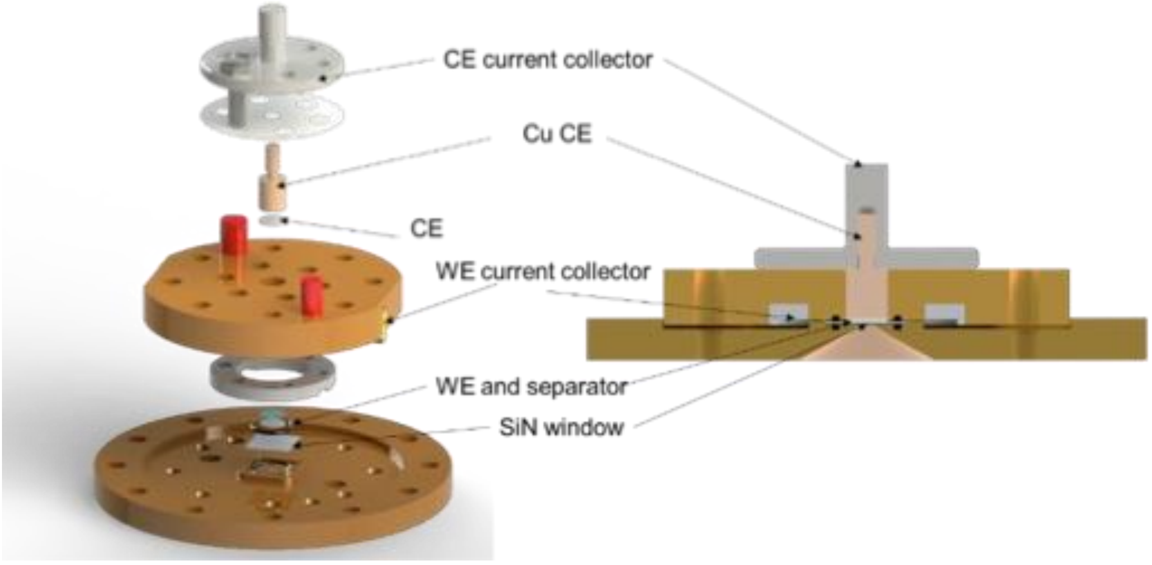


**Figure S4** Detailed sketch of static cell presented in figure 3 of the main text.

## S4. X-ray filters installed at the OÆSE endstation

The following filters have been installed in the beamline (after the Au mesh used as beam monitor)

- 1 um SiN membrane (for soft x-ray)
- 100 nm SiC membrane + 40 nm sputtered Ir (for soft x-ray)
- 12 um Kapton foil (for hard x-ray)

- 24 um Kapton foil (for hard x-ray)
- 36 um Kapton foil (for hard x-ray)

The calculated transmission of the filters installed is depicted in **Figure S5**.

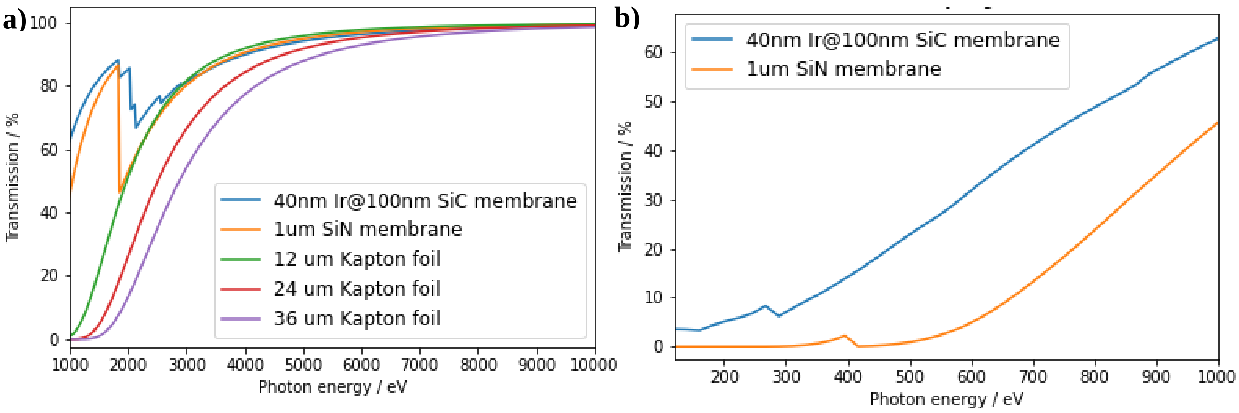


**Figure S5** Calculated transmission of the attenuation filters installed in the *operando* beamline section at the **a)** tender/hard and **b)** soft X-ray regimes.

### S5. Calculation of the flux as a function of undulator gap

The photocurrent (proportional to photon flux) for different undulator gap values around the optimized undulator gap configuration has been measured at the last focusing mirror (M4) and at the sample's position with a photodiode. Measurements were performed in the O K-edge and Ni $L_{23}$-edge region of the UE48 soft x-ray beamline using an 800 l/mm planar grating monochromator and the 3rd undulator's harmonic. **Figure S6** shows these measurements, with the undulator gap offset as the independent variable. Undulator gap offset ($ID_{offset}$) is an empirical parameter introduced as follows in the calculation of the target energy used to tune the undulator gap:

$$E_{target}\ (eV) = E_{set}\ (eV) * ID_{slope} + ID_{offset}\ (eV)$$

The $E_{target}$ value is then used to assign an undulator gap value as previously optimized.

The photocurrent at the central energy of these regions (540 eV and 860 eV for the O K-edge and Ni $L_{23}$-edge respectively) normalized to the maximum flux is plotted here as a function of the undulator gap offset $ID_{offset}$, with 0 offset corresponding with the optimized undulator gap's value corresponding to a maximum flux, i.e. intensity of 1. A polynomial fit to the measured flux values at 540 and 860 eV enables the extrapolation of the photon flux calculation to a broader x-ray range of 400 to 1000 eV as observed in **Figure S7** and **Figure 5a** of the main text. This allows the prediction of the flux as a function of the undulator gap for every energy between 400 and 1000 eV with the same beamline configuration that these measurements (800 l/mm and 3rd undulator's harmonic). Due to the small differences in undulator gap values for different photon flux (less than 1 mm in the range 400-1000

eV), **Figure 5a** of the main text reproduces the results of figure S7 but depicting instead the undulator gap's shift to the optimized configuration (100% flux).

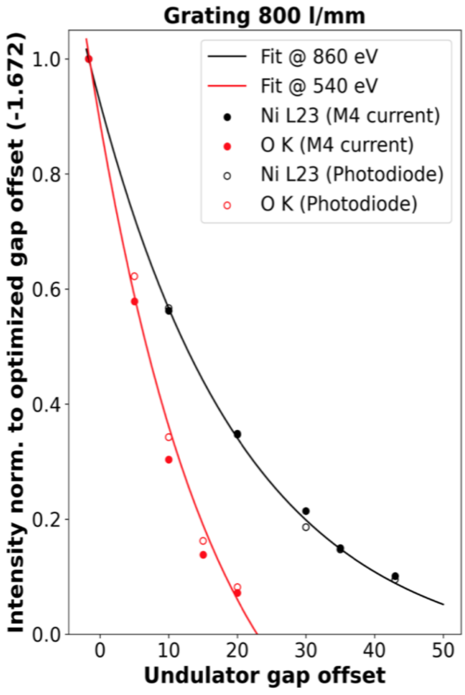


**Figure S6** *Flux prediction at OÆSE endstation as a function of undulator gap.* In black, intensity measured at an excitation energy of 860 eV (Ni $L_{23}$-edge), while red circles correspond to measurements at 540 eV (O K-edge). Filled circles are measured at the last optical component (focusing mirror, "M4 current") and empty circles are measured at the sample position of the endstation ("Photodiode"). A polynomial curve has been fitted to both measured intensities ("M4 current" and "Photodiode") with comparable Pearson correlation parameters.

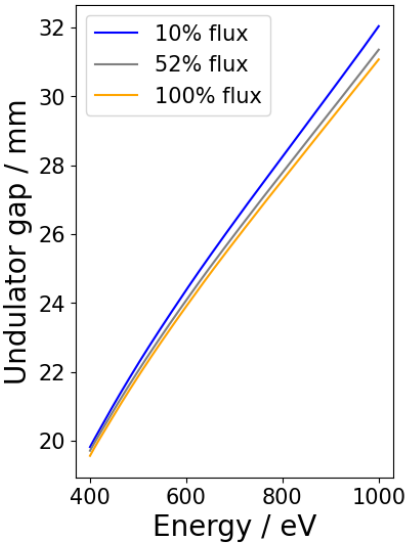


**Figure S7** *Undulator gap prediction*: Calculation of the undulator gap as function of energy for 3 different photon fluxes (10%, 52% and 100% of the optimized undulator gap configuration). The values of the undulator gap can be predicted by extrapolating the values measured at 540 and 860 eV, as explained in the text.

## S6. *cff* detuning

The *cff* (critical focusing condition) can be adjusted around the optimum value (trade-off between photon flux, spatial resolution and resolving power) to minimize the incoming photon flux at the sample position. As depicted in **Figure S8** for a photon energy of 550 eV, the detuning of the *cff* parameter can translate into a flux reduction of 50% in comparison with the optimized beamline configuration at *cff* = 2.25. Though no noticeable loss of resolving power was observed in the range between 1.45 and 2.80, it can be expected that the extreme detuning of the *cff* parameter drastically affects the beam profile and its energy resolution.

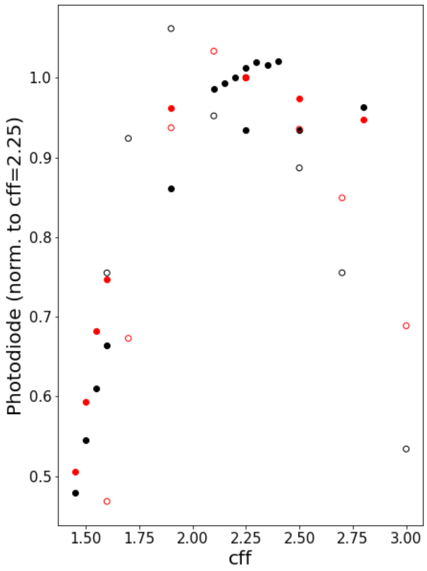


**Figure S8** *cff detuning*: Photon flux measured at a photon energy of 540 eV (red) and 860 eV (black) with a photodiode located after the last optical element. Filled circles are measured using a 800 l/mm planar grating monochromator, while empty circles correspond to 400 l/mm (both employing the 3rd undulator's harmonic). By decreasing the *cff* to values below 1.5, a loss of photon flux of around 50% was observed.

## S7. Monitoring the in-situ growth of an electrodeposited Cu-based material by soft XAS

Copper plating is a well-established electrodeposition process with significant industrial relevance. Its applications span from microelectronics ***[Liu et al., 2010]*** to the production of widely spread alloys like brass or bronze ***[Kamel et al., 2022]*** or fabrication of sensors, like glucose biosensors ***[Naikoo et al., 2022]***. Though previous publications have delved on the electrochemical mechanisms governing copper electrodeposition ***[Velasco-Velez et al., 2018]***, a knowledge gap persists regarding the precise function of intermediates in the reaction and about the specific electrochemical conditions that favor the deposition of a certain stable chemical species, as well as about the growth and nucleation behavior of the electrodeposited film [***Grujicic & Pesic, 2002***].

XAS can yield insights into the chemical differences between the electrode/electrolyte interface and the structure of the copper electrodeposited materials owing to the distinct probing depths of the photon energies employed, from the more surface sensitivity of the soft X-rays to the larger information depth of the hard X-rays.

In **Figure S9a**, the characteristic cyclic voltammogram of the Pt | aq. 0.1 M $CuSO_4$ electrochemical system is shown, where two main operating regions can be found. At potentials below ~0 $V_{SSC}$, a region of reduction is observed, where typically the cupric ions in the electrolyte undergo electroplating under a 2-electron process to produce a metallic Cu film, as described by the following equation:

$$Cu^{2+} + 2e^{-} \rightarrow Cu_{(s)}$$

However, the appearance of $Cu^{+}$ species in the form of a stable $Cu_2O$ solid has also been reported during the electrodeposition of copper in acidic media at these reducing potentials, as a result of the cuprous ion reactions with water ***[Velasco-Velez et al., 2018]***:

$$Cu^{2+} + e^{-} \rightarrow Cu^{+}$$

$$2Cu^{+} + H_2O \rightarrow Cu_2O_{(s)} + 2H^{+}$$

The equilibrium between Cu(0) and Cu(I) species in solid form is highly dependent of the electrodeposition conditions , e.g. electrolyte pH or applied potential, as suggested by the intricate Cu-water Pourbaix diagram ***[Celante & Freitas, 2010]***. A two-step deposition process with a $Cu^{+}$-based intermediate has also been proposed elsewhere ***[Ghosh et al., 2019]***.

Secondly, an oxidizing region can be found for applied potentials higher than ~0 $V_{SSC}$, where the solid Cu film tends to dissolve back to solvated $Cu^{2+}$ ions. This effect is manifested in **Figure S9a** for potentials higher than 0.4 $V_{SSC}$, where the very low current density $j_{geo}$ measured can be attributed to the total dissolution of solid Cu. The focus of this study lies on the reductive region of the voltammogram, aiming at understanding the competing mechanisms occurring at the electrodeposition process of a Cu film by *in-situ* soft and hard XAS.

For the proof-of-concept experiment, the electrodeposition of Cu has been achieved by applying a constant reductive potential (< 0 $V_{SSC}$) on the WE for a certain amount of time. The chronoamperometric (CA) protocol used for electrodepositing Cu in the experiment presented in this subsection employed a reductive potential of -0.1 $V_{SSC}$ for 15 minutes, with OCP pauses after 1, 5, 10 and 15 minutes to record the spectra. As displayed in the inset of **Figure S9a**, an accumulated charge density of -0.3 C $cm^{-2}$ was passed after 15 minutes at an average current density $\langle j_{geo} \rangle$ of -0.33 mA $cm^{-2}$.

As described in **section §8 of the Supplementary Information**, the equivalent amount of electrodeposited Cu can be derived from the charge passed during the electrochemical reaction based on Faraday's law, assuming that all current passed is employed in the reduction process (100% Faradaic efficiency). The deposition rate, under the assumption of a uniform and dense $Cu_2O$ film, as it will be further discussed later, is estimated at approximately 25 nm per minute. Given the deviation of the electrodeposition from an ideal layer-by-layer growth, we adopt an "equivalent thickness"

parameter to describe the amount of electroplated Cu. Importantly, the equivalent thickness demonstrates a linear correlation with both electrodeposition time and charge density, as depicted in the inset **Figure S9a**.

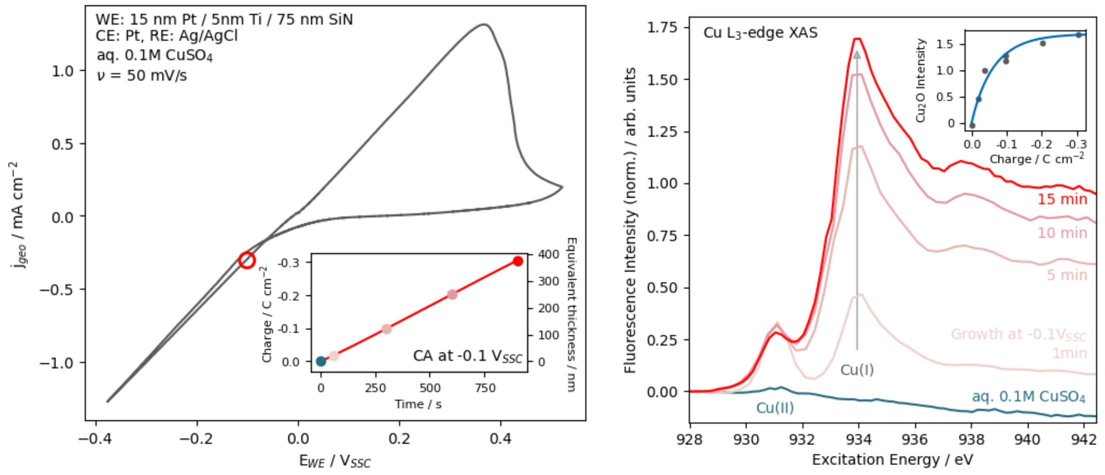


**Figure S9** **a)** Cyclic voltammogram of the Pt | aq. 0.1M $CuSO_4$ electrochemical system. Inset illustrates a chronocoulometric measurement at -0.1 $V_{SSC}$ for 15 min (<$j_{geo}$> = -0.33 mA $cm^{-2}$) with the calculated deposited film thickness on the secondary *y* axis (assuming a pure Faradaic process, a homogeneous film and using the theoretical solid $Cu_2O$ density). **b)** X-ray absorption spectra at the Cu $L_3$-edge in TFY mode for different electrodeposition times at -0.1 $V_{SSC}$ until 15 min, with the spectrum of the aq. 0.1 M $CuSO_4$ electrolyte on the bottom as a reference of the system before electrodeposition. Inset depicts the fluorescence intensity of the peak associated with the growing $Cu_2O$ film (at 934.0 eV) as a function of the passed charge density. The gray line depicts an $A(1-e^{-B*t})$ function, highlighting the non-linear growth behavior of the spectroscopic signal due to X-ray intensity attenuation.

To gain more insights into the growth of the film as a function of the electrodeposition time, X-ray absorption spectra collected at the Cu $L_3$-edge in TFY mode at different deposition times are presented in **Figure S9b**. As detailed before, the film was electrodeposited for 15 min, until the material reached an equivalent film thickness of 370 nm, where most of the photons arising from the interface of the electrode with the electrolyte are already adsorbed by the bulk of material before reaching the fluorescence detector. At this equivalent thickness and assuming an uniform film, the photon transmission is negligible (0.4% at 940 eV, as discussed in **Section §10 of the Supplementary Information**) and further growth of material would not contribute significantly to the $Cu_2O$ spectroscopic signal, which is consistent with the trend towards the saturation in the Cu(I) signal, as seen in the inset of **Figure S9b**.

Already after 1 min of electrodeposition, a clear intensity increase of the peak at 934.0 eV is observed in **Figure S9b**, while the spectroscopic feature at 931.1 eV associated with the Cu(II) species initially

present as cations in the $CuSO_4$ electrolyte increases only slightly. The peak at 934.0 eV is typically related to both Cu(I) and Cu(0) species ***[Velasco-Velez et al., 2018]***, but the spectral features at higher excitation energies suggest that the electrodeposited species in these conditions is Cu(I) ***[Weatherup et al., 2018]*** in the form of solid $Cu_2O$, as has been reported previously for similar electrochemical conditions (aq. 0.005 M $CuSO_4$ in ***[Velasco-Velez et al., 2018]***). The remaining Cu(II) spectroscopic feature after the thin film growth might be attributed to the presence of the solvated Cu(II) species in the interface, i.e. double layer. The calibration of the excitation energy has been performed following the protocol described in **section §9 of the Supplementary Information.**

Intuitively, the spectral features related to the electrodeposited $Cu_2O$ film increase monotonically as a function of the electrodeposition time and after 15 minutes of growth the spectroscopic contribution of the $Cu_2O$ film totally dominates the spectrum. As a rough quantification of this growth, the inset of **Figure S9b** depicts the fluorescence intensity of the peak at 934.0 eV associated with the growing $Cu_2O$ film as a function of the passed charge density, i.e. electrodeposition time. Interestingly, the increase of the spectroscopic signal of the electrodeposited film does not correlate linearly with the passed charge, in contrast with the linear growth of the material observed in **Figure S9a**. This is explained by the exponential behavior of the attenuation of X-ray intensity through a solid material like the growing $Cu_2O$ sample. As detailed in **Section §10 of the Supplementary Information**, the intensity of the fluorescence signal arising from a uniform film with thickness *t* contributes to the spectrum by:

$I(t) = \frac{I_0}{2\mu}(1 - e^{-2\mu \cdot t})$, where $I_0$ is the intensity of the infinitely thick $Cu_2O$ film and μ is the absorbance of the material. As a guide-to-the-eye, an $A(1\text{-}e^{-B*t})$ function has been plotted in the inset of **Figure S9b**, reproducing the expected intensity attenuation as a function of the film thickness.

### S8. Calculation of electrodeposited $Cu_2O$ equivalent thickness

When depositing $Cu_2O$, the relevant chemical reaction at the electrode is the reduction of copper ions ($Cu^{2+}$) to form copper (I) oxide ($Cu_2O$). The balanced half-reaction for this process is:

$$Cu^{2+} + e^- \rightarrow Cu^+$$

$$2Cu^+ + H_2O \rightarrow Cu_2O_{(s)} + 2H^+$$

In this reaction, two electrons are transferred for each copper ion reduced. This means that *z* in Faraday's law equation will be 2.

For $Cu_2O$:

Molar mass *M* = 143.09 g/mol

Physical theoretical density at room temperature *rho* = 6.00 g/cm$^3$

The total charge passed (*Q*) is given by $Q=I \cdot t$, while the mass of $Cu_2O$ deposited (*m*) is given by:

$$m = M*Q / (z * F)$$

where *F* = 96485 C/mol

The equivalent thickness (*t*) of the electrodeposited $Cu_2O$ can be calculated by:

$$t = m/(rho * Area)$$

where the available geometric area of the electrode in the 3-electrode flow-through used in the OÆSE endstation is 0.503 $cm^2$.

## S9. Energy calibration at the Cu $L_3$-edge

The energies used for calibration were obtained from the calculated atomic K edge of silicon and the energy difference between Si and SiN reported in Chang *et al.* (1999) and Owens & Fraser (2002), detailed in the following (in parenthesis, 2$^{nd}$ order energies of the Si K-edge arising before the Cu $L_3$-edge):

$$E(Si) = 1838.9 \text{ eV } (919.45 \text{ eV})$$

$$E(SiN) = E(Si) + 4.5 \text{ eV} = 1843.4 \text{ eV } (921.7 \text{ eV})$$

For the measurements presented in section 4 of the main text, the energy shift in the Cu $L_3$-edge is 2.45 eV. The **Figure S10** depicts the XAS of the $SiN_x$ window, where the uncalibrated excitation energy has been converted to 2$^{nd}$ order energies for comparison purposes:

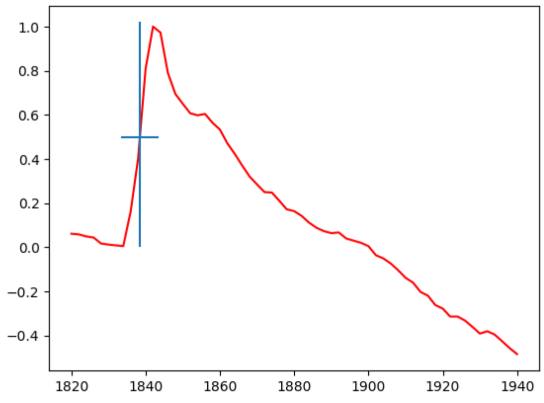


**Figure S10** *Si K-edge XAS measurements on a SiNx window*. In the x-axis, the uncalibrated excitation energy has been converted to 2nd order energies.

## S10. Calculation of X-ray intensity attenuation in an electrodeposited Cu thin film

In a photon-in/photon-out experiment, the intensity of the fluorescence photons arising from a section of $Cu_2O$ at a distance *x* of the thin, semi-transparent x-ray window is given by:

$$I(x) = I_0 e^{-2\mu \cdot x}$$

where $I_0$ is the incident photon intensity and μ is the photoabsorption cross-section of the material; $Cu_2O$ in this case. As expected, the farther away the origin of the photons from the window is, less intensity will contribute to the spectroscopic signal.

To understand how the spectroscopic signal changes as a function of the thickness of the electrodeposited Cu film, an integral between 0 and the thickness (t) is necessary:

$$I(t) = \int_0^t I_0 e^{-2\mu \cdot x} dx = \frac{I_0}{2\mu}(1 - e^{-2\mu \cdot t})$$

To reproduce the analytical behavior observed in the fluorescence intensity of the electrodeposited $Cu_2O$ as a function of its thickness (i.e. charge applied during electrodeposition in C $cm^{-2}$), a guide-to-the-eye function of the following form has been implemented:

$$I(t) = A(1 - e^{-B \cdot t})$$

with parameters *A* and *B* adjusted to the empirical values found in this experiment.

As observed in **Figure S11**, a drastic increase of the spectroscopic signal *I(t)* is expected for the first nanometers of electrodeposited $Cu_2O$, while the thicker the material becomes, smaller increase of the signal is observed:

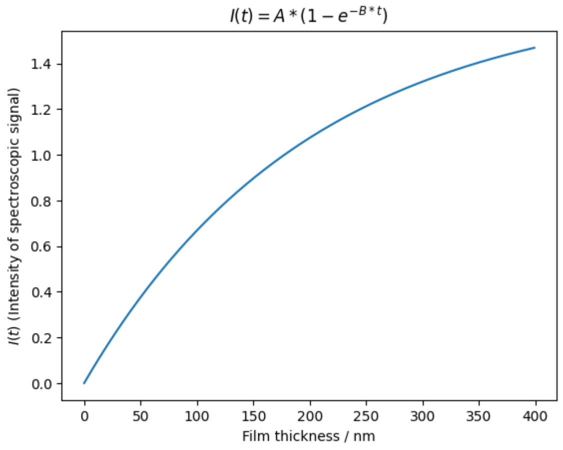


**Figure S11** *Calculated fluorescence intensity of a $Cu_2O$ compact film as a function of its thickness.* The A(1-exp(-B*t) ) function shows the expected spectroscopic intensity as a function of the film thickness.

The material was grown until the thickness of the $Cu_2O$ film absorbs most of the photons arising from the interface of the electrode with the electrolyte.

Maximum growth of the $Cu_2O$ film achieved in the experiment is 370 nm. Considering that photons travel 2 times through the material (exciting photon (in) & fluorescence photon (out)), the collected photons contributing to the spectroscopic intensity travel 740 nm. At this thickness, photon transmission at 940 eV (after the edge Cu $L_3$-edge) is only 0.4%.

### S11. Chronoamperometric profiles of the two electrodeposited Cu materials studied by sXAS and hXAS

The two *in-situ* grown Cu samples discussed in the main text were electrodeposited in an aq. 0.1M $CuSO_4$ electrolyte onto a 15 nm Pt electrode with a constant potential. Below, the current profiles of the 2 electrodeposition processes.

The electrodeposited samples were grown under an average current density of -0.06 mA cm$^{-2}$ and -0.09 mA cm$^{-2}$ for the studies with and without 30 sec OCP pauses respectively, 20-30% of the current passed for the experiment shown in in **Section §6 of the Supplementary Information**.

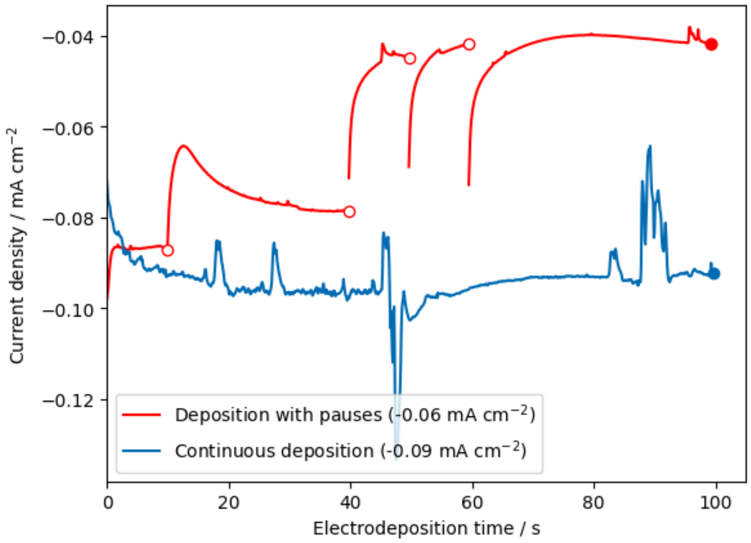


**Figure S12** *Chronoamperometric profiles of the 2 electrodeposited samples discussed in the main text.* CA experiments of *in-situ* grown Cu materials, deposited "continuously" (blue) and including intermittent potential application, labelled "deposition with pauses" (red).

### S12. SEM micrographs of electrodeposited Cu samples at different conditions

Three different electrodeposited Cu samples were prepared on a 15nm Pt electrode in an aq. 0.1M $CuSO_4$ electrolyte with the following conditions:

A) *Deposition with pauses*: 1 min electroreduction + 30 sec OCV + 9 min electroreduction (reducing potential: $E_{we}$ = -0.1 $V_{RHE}$)

B) *Continuous deposition:* 10 min electroreduction (reducing potential: $E_{we}$ = -0.1 $V_{RHE}$)

C) *Very reductive:* 4 min electroreduction (reducing potential: $E_{we}$ = -0.3 $V_{RHE}$)

The different morphologies and Cu coverages can be observed in the following micrographs measured using a Phenom Pharos G2 Desktop FEG-SEM with a voltage of 5kV or 15kV with an Energy-dispersive X-ray spectroscopy (EDX) option.

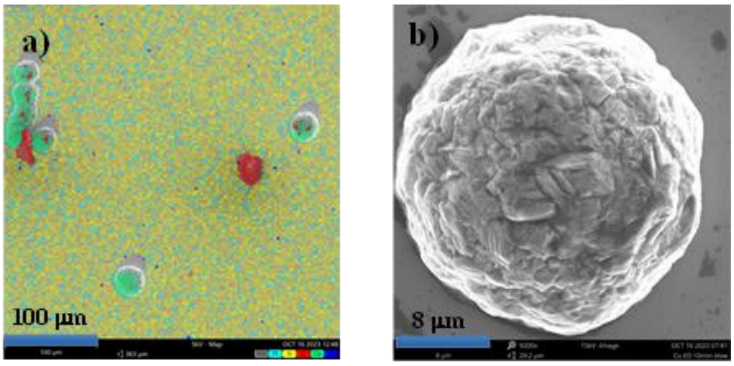


**Figure S13** *Deposition with pauses*: **a)** 5kV EDX map showing partial Cu coverage (green) of the 15 nm Pt (yellow) electrode deposited on a Si substrate (blue) with a 5 nm Ti interlayer, **b)** 15kV image showing the typical morphology of the Cu structures grown on Pt. Structure sizes range between 20 and 30 μm.

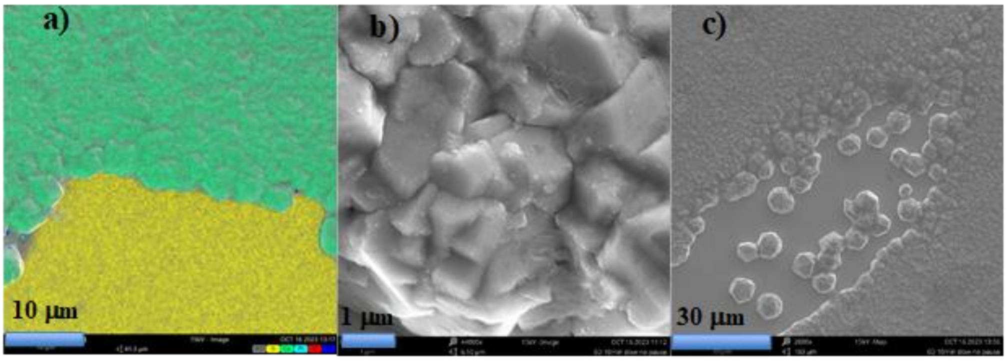


**Figure S14** *Continuous deposition*: **a)** 15kV EDX map showing partial Cu coverage (green) of the 15 nm Pt (yellow) electrode deposited on a Si substrate (blue) with a 5 nm Ti interlayer, **b)** 15kV image showing the typical morphology of the Cu structures grown on Pt, where crystallites size is around 1 μm and form a cohesive network decorated by smaller structures of <100 nm sizes, **c)** 15kV image showing other Cu structures grown on the edges of the cohesive network, where particles show sizes around 5-10 μm.

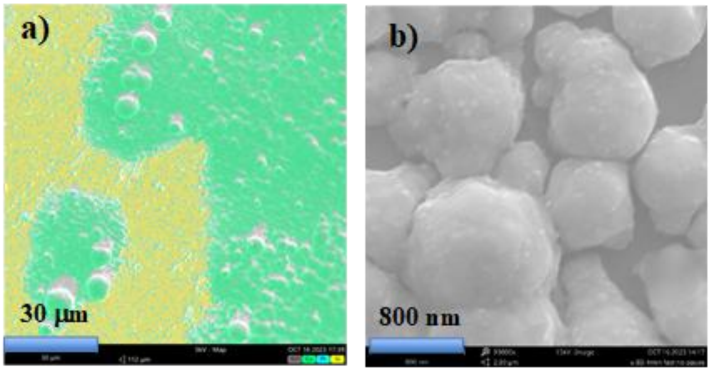


**Figure S15** *Very reductive*: **a)** 15kV EDX map showing partial Cu coverage (green) of the 15 nm Pt (yellow) electrode deposited on a Si substrate (blue) with a 5 nm Ti interlayer, **b)** 15kV image showing the typical morphology of the Cu structures grown on Pt, where crystallites around 1 µm big form a cohesive network decorated by smaller structures of <100 nm sizes

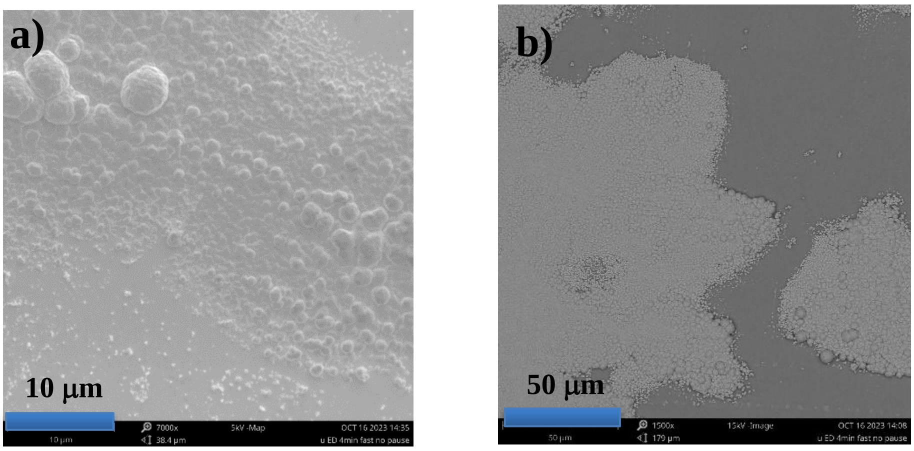


**Figure S16** Partial coverage of an electrodeposited material under *very reductive* conditions: **a)** 5kV and **b)** 15kV image show the partial coverage of the electrode even at these conditions. Moreover, other Cu structures smaller than 3 µm in size grow on the edges of the cohesive network.

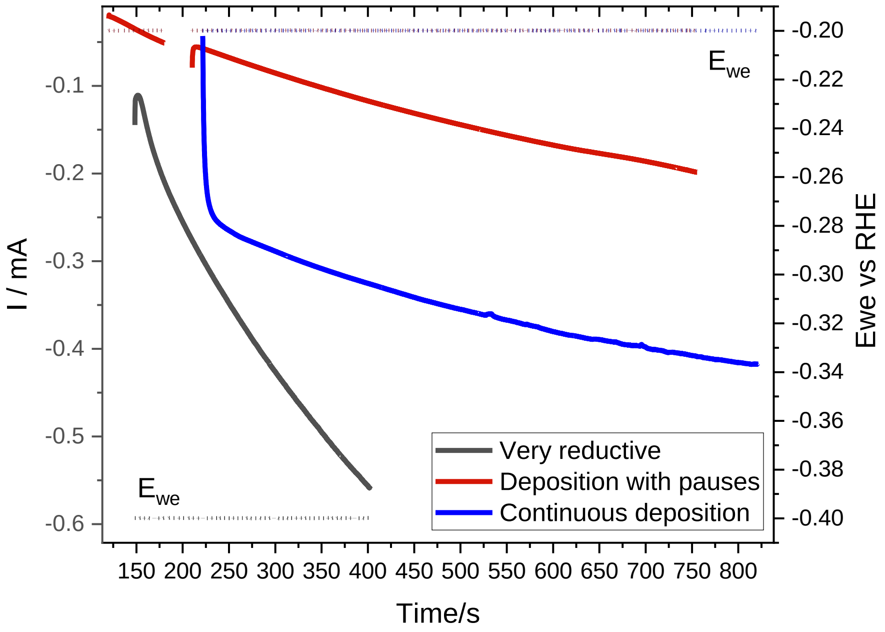


**Figure S17** Electrodeposition of the 3 Cu samples measured by SEM. Chronoamperometries for the samples *Deposition with pauses* (red) *Continuous deposition* (blue) and *Very reductive* (black). Current is not normalized to surface area of the electrode (15 nm Pt on 5 nm Ti deposited by magnetron sputtering on a Si substrate)

### References of supporting information

Celante V. G. and Freitas, M. B. J. G. (2010). *J. Appl. Electrochem* **40**, 233.

Chang, Y. K., Hsieh, H. H., Pong, W. F., Tsai, M. H., Dann, T. E., Chien, F. Z. & Chen, Y. F. (1999). *J. Appl. Phys.* **86**, 5609.

Ghosh, R., Sudha, V. & Harinipriya, S. (2019). *Bull. Mater. Sci.***42**, 43.

Grujicic, D. & B. Pesic, B. (2002). *Electrochimica Acta*, **47**, 2901.

Kamel, M. M., Alzahrani, E., Abbass, A. M. & Ellah, A. A. A. (2022). *Biointerface Res. Appl. Chem.* **13**, 157.

Liu, Y., Yin, L., Bliznakov, S., Kondos, P., Borgesen, P., Henderson, D. W., Parks, C., Wang, J., Cotts, E. J. & Dimitrov, N. (2010). *IEEE Trans. Compon. Packaging Technol.* **1**, 127.

Naikoo, G A., Awan, Z., Salim, H., Arshad, F., Hassan, I. U., Zamani Pedram, M., Ahmed, W., Faruck, H. L., Aljabali, A. A. A., Mishra, V., Serrano-Aroca, A., Goyal, R., Negi, P., Birkett, M.,

Nasef, M. M., Charbe, N. B., Bakshi, H. A. & Tambuwala, M. M. (2022). *Bioeng. Translat. Med.* **7**, e10248

Owens, A., Fraser, G. W. & Gurman, S. J. (2002). *Radiat. Phys. Chem.* **65**, 109.

Velasco-Vélez, J.-J., Skorupska, K., Frei, E., Huang, Y.-C., Dong, C.-L., Su, B.-J., Hsu, C.-J., Chou, H.-Y., Chen, J.-M., Strasser, P., Schlögl, R., Knop-Gericke, A. & Chuang, C.-H. (2018). *J. Phys. Chem. B*, **122**, 780.

Weatherup, R. S., Wu, C. H., Escudero, C., Pérez-Dieste, V. & Salmeron, M. B. (2018). *J. Phys. Chem. B*, **122**, 737.
